# Supplementary material for: Factors influencing participation in randomised clinical trials among patients with early Barrett’s neoplasia: a multicentre interview study
Source: BMJ Open. 2023 Jan 6;13(1):e064117. doi: 10.1136/bmjopen-2022-064117 (PMC9827249; doi:10.1136/bmjopen-2022-064117)
Supplement: Supplementary data [file bmjopen-2022-064117supp001.pdf]

Supplementary material A – Interview Topic Guide

This guide is designed to be used flexibly to encourage patients to talk about what matters to them. If the patient brings up an issue not covered here, this will be actively explored. The content is led by the patient, the interviewer's role is to prompt in a way that helps to address the research focus, which is to identify and understand the nature of barriers and enablers to participation in studies of this kind.

The layout of the topic guide may also be amended to suit the individual doing the interviews.

Preliminaries, not necessarily in this order, to be determined by interviewer:

Check that timing of phone call is still convenient.

Introduce interviewer.

Go through information sheet; give opportunity to ask questions, especially confidentiality, anonymity issues, audio recording and transcribing (why it is important).

Confidentiality – what you say will not get back to the center treating you; interviews will be analysed all together, all identifying details removed, and themes summarised. For instance, we might find that several people mention a particular thing – for instance there might be an issue about not feeling rushed – and we will report that; but we would not mention your name or the names of doctors or nurses.

The only situation in which I would break confidentiality is if I heard that someone's life was in danger – this is most unlikely in an interview like this.

Talk through nature of interview – i.e. I am interested in your views so there are no wrong or right answers. I have a list of topics, which is here just to start off the conversation, but what we are really interested in is what matters to you.

Can stop at any time, just ask;

Can say you'd prefer to move on to the next question if there is something that it's not convenient to talk about at the moment

We can take as long as you like, or stop at a particular time – do you have a particular time you need to finish?

Any questions?

We are interested in how people feel about taking part in studies of the sort of treatment you have been having, so we are talking to you because you were asked to take part in a study to do with the treatment of your gullet. Can I check first, is that right?

DECIDING

Some people make up their minds really quickly about taking part in research. Others prefer to weigh things up for longer. How tricky was it for you to decide?

What sorts of things led to your decision?

Prompts

WHO AND HOW recruited

Did it matter WHO asked you, or would you have just said yes/no anyway?

Did it matter HOW it was explained, how much information did you want, what sort of information (explore role of written information, opportunities to talk and ask questions), or would you have said yes anyway …

SHARED OR SOLE DECISION

Many people see this sort of thing as solely their own decision, others make a decision but also like to discuss it with family or friends, how did you feel? (did you talk to partner/family/others about it – tell me a bit more about that)

How did partner/family/others feel about you taking part, did that make a difference to you?

If applicable – how much information did you husband/wife/family want? Who explained it to them? How well did this work out do you think?

PERCEPTION OF AIM AND STRUCTURE OF STUDY

I wondered how it feels to be a patient – were there parts of the study you understood better than others? Did you feel as if you understood what the study aims to do? Can you tell me about that? Were there some things that are harder to understand? (For you, or maybe even if it was OK for you, were there parts that might be hard for other patients). How does it feel to be in the study? How do you think you'd feel if you'd decided against it?

Was there anything that made it easy for you to decide?

Was there anything that made it difficult to decide?

LOOKING BACK:

Looking back, do you feel positive about deciding (not) to take part, or are there some things you are less positive about? (or if you prefer not to look back that's fine, just say so) – What has your treatment been like? How did it feel? Do you know what the treatment was? How was your treatment decided?

If participated: We hope that this didn't happen, but did you at any point feel like dropping out of the study? Tell me about that (what meant that you carried on, how difficult was it, did someone or something make a difference, was there anything someone could have done that would have made that easier etc.)

IF PARTICIPATED: VIEWS ON TAKING PART:

In your view, what have been the good things and the less good things about taking part in the study? Take them in whatever order suits you.

Keep prompting until covered

VIEWS ON TREATMENT:

And in a similar vein, (if not already covered), what have been the good points and the bad points about the treatment you have been receiving? Feel free to mention anything even if it seems minor, because it's important to know what it is like for patients – you are the only expert at being on the receiving end.

Are there any things that you think would help with future studies –?

Things that would help people to decide whether or not to take part

Things that would help people to carry on /not 'drop out',

FUTURE TRIALS:

Now I'd like to ask a few final questions. The treatment centers involved in this study would like to do another piece of research, comparing endoscopic treatment to surgery. We are NOT asking you to take part in this. The reason I am asking you about it is to get your advice because you have some experience of what it's like to be in a study.

Imagine patients were told that there was a need to compare a surgical treatment with an endoscopic treatment because although both seem roughly equivalent, in terms of success, we don't know all their advantages and disadvantages. Taking part would not benefit the patients themselves, but it would provide information that would help in treating people in the future.

How do you think people would feel to be asked whether they were be willing to receive either surgery, or endoscopic treatment, at random (i.e. they would have a fifty fifty chance of each treatment – like deciding by tossing a coin)?

What sorts of things would they want to know before they decided? (explore)

What sorts of things might put them off taking part? (explore)

What sorts of things might mean they were likely to take part? (explore)

Any general messages for people who design and run studies? (i.e. things that would make life easier for those being asked to take part, things that some centers do really well when it comes to research, that other doctors could learn from – or the opposite)

Anything else you want to say about taking part in this type of research? For instance is there anything that makes research into this condition (gullet-related) different to research in other conditions? Tell me a bit about it.

Last - What do you think researchers should be trying to achieve in the future? What should they be looking at, to help with this condition?

Is there anything else you want to say, or to ask me? I cannot answer questions about your particular treatment but I can pass on queries.

Give contact details in case participant wishes to add or retract anything

Thanks
