# Supplementary material for: Factors influencing participation in randomised clinical trials among patients with early Barrett’s neoplasia: a multicentre interview study
Source: BMJ Open. 2023 Jan 6;13(1):e064117. doi: 10.1136/bmjopen-2022-064117 (PMC9827249; doi:10.1136/bmjopen-2022-064117)
Supplement: Author's manuscript [file bmjopen-2022-064117.draft_revisions.pdf]

BMJ Open is committed to open peer review. As part of this commitment we make the peer review history of every article we publish publicly available.

When an article is published we post the peer reviewers' comments and the authors' responses online. We also post the versions of the paper that were used during peer review. These are the versions that the peer review comments apply to.

The versions of the paper that follow are the versions that were submitted during the peer review process. They are not the versions of record or the final published versions. They should not be cited or distributed as the published version of this manuscript.

BMJ Open is an open access journal and the full, final, typeset and author-corrected version of record of the manuscript is available on our site with no access controls, subscription charges or pay-per-view fees (http://bmjopen.bmj.com).

If you have any questions on BMJ Open's open peer review process please email info.bmjopen@bmj.com

BMJ Open

Factors influencing participation in randomised clinical trials among patients with Barrett's early neoplasia: A multi-centre interview study

Journal:	*BMJ Open*
Manuscript ID	bmjopen-2022-064117
Article Type:	Original research
Date Submitted by the Author:	22-Apr-2022

Complete List of Authors:	Peerally, Mohammad; University of Leicester,<br>Jackson, Clare; University of Nottingham Faculty of Medicine and Health Sciences<br>Bhandari, P; Queen Alexandra Hospital, Portsmouth<br>Ragunath, Krish; Curtin University,<br>Barr, Hugh; Gloucestershire Royal Hospital<br>Stokes, Clive; Gloucestershire Royal Hospital<br>Haidry, Rehan; University College Hospital<br>Lovat, Laurence; University College London, Division of Surgery and Interventional Science<br>Smart, Howard; Royal Liverpool Hospital, Department of Gastroenterology<br>De Caestecker, John; The University of Leicester; University Hospitals of Leicester NHS Trust

Keywords:	Endoscopy < GASTROENTEROLOGY, Oesophageal disease < GASTROENTEROLOGY, Adult gastroenterology < GASTROENTEROLOGY

SCHOLARONE™ Manuscripts

I, the Submitting Author has the right to grant and does grant on behalf of all authors of the Work (as defined in the below author licence), an exclusive licence and/or a non-exclusive licence for contributions from authors who are: i) UK Crown employees; ii) where BMJ has agreed a CC-BY licence shall apply, and/or iii) in accordance with the terms applicable for US Federal Government officers or employees acting as part of their official duties; on a worldwide, perpetual, irrevocable, royalty-free basis to BMJ Publishing Group Ltd ("BMJ") its licensees and where the relevant Journal is co-owned by BMJ to the co-owners of the Journal, to publish the Work in this journal and any other BMJ products and to exploit all rights, as set out in our licence.

The Submitting Author accepts and understands that any supply made under these terms is made by BMJ to the Submitting Author unless you are acting as an employee on behalf of your employer or a postgraduate student of an affiliated institution which is paying any applicable article publishing charge ("APC") for Open Access articles. Where the Submitting Author wishes to make the Work available on an Open Access basis (and intends to pay the relevant APC), the terms of reuse of such Open Access shall be governed by a Creative Commons licence – details of these licences and which Creative Commons licence will apply to this Work are set out in our licence referred to above.

Other than as permitted in any relevant BMJ Author's Self Archiving Policies, I confirm this Work has not been accepted for publication elsewhere, is not being considered for publication elsewhere and does not duplicate material already published. I confirm all authors consent to publication of this Work and authorise the granting of this licence.

For peer review only

Factors influencing participation in randomised clinical trials among patients with Barrett's early neoplasia: A multi-centre interview study

For peer review only

Authors

Mohammad Farhad Peerally PhD[1,2] , Clare Jackson MSc[3], Pradeep Bhandari MD[4], Krish Ragunath MD[5], Hugh Barr MD[6], Clive Stokes MD[6], Rehan Haidry MD[7], Laurence B Lovat PhD[7,8], Howard Smart MD[9], John S de Caestecker MD[10,11]

Author emails:

mfp6@le.ac.uk (corresponding author)

Clare.Jackson@nottingham.ac.uk

pradeep.bhandari@porthosp.nhs.uk;

krish.ragunath@curtin.edu.au

hugh.barr@glos.nhs.uk

clivestokes@nhs.net

rehan.haidry@nhs.net>

l.lovat@ucl.ac.uk

howard.smart@rlbuht.nhs.uk

john.decaestecker@uhl-tr.nhs.uk

Affiliations

1. Digestive Diseases Unit, Kettering General Hospital, Rothwell Rd, Kettering NN16 8UZ, UK.
2. SAPPHIRE, Department of Health Sciences, College of Life Sciences, George Davies Centre, University of Leicester, University Road, Leicester, LE1 7HA, UK.
3. School of Medicine, University of Nottingham, Queen's Medical Centre, Nottingham, NG7 2UH, UK.
4. Department of Gastroenterology, Queen Alexandra Hospital, Southwick Hill Road, Portsmouth, PO6 3LY, UK.
5. NIHR Nottingham Biomedical Research Centre, Nottingham University Hospitals NHS Trust and the University of Nottingham, Derby Road, Nottingham, NG7 2UH, UK.
6. Department of Surgery, Gloucester Royal Hospital, Great Western Road, Gloucester, Gloucestershire, GL1 3NN, UK.
7. Department of Gastroenterology, University College Hospital, 235 Euston Road, London, NW1 2BU, UK.

For peer review only

8. Division of Surgery and Interventional Science, University College London, Gower Street, London WC1B 6BT
9. Department of Gastroenterology, Royal Liverpool Hospital, Prescot St, Liverpool L7 8XP, UK.
10. Leicester Cancer Research Centre, College of Life Sciences, The University of Leicester, University Road, Leicester, LE1 7RH, UK.
11. Digestive Diseases Centre, University Hospitals of Leicester NHS Trust, Infirmary close, Leicester, LE1 5WW, UK.

Corresponding author

Dr Mohammad Farhad Peerally (mfp6@le.ac.uk)

Room 4.33, George Davies Centre, Department of Health Sciences, College of Life Sciences, George Davies Centre, University of Leicester, University Road, Leicester, LE1 7HA, UK.

Phone: 07977764607

Running head

Influencers to participation in endoscopy RCT

Word count

3955 words

Grant support

This article presents independent research funded by the National Institute for Health Research (NIHR) under its Research for Patient Benefit Programme (grant no. PB-PG-0711-25066).

For peer review only

Trial registration

ClinicalTrials.gov Identifier: NCT01733719

Disclosure

The views expressed are those of the authors and not necessarily those of the NHS, the NIHR, or the Department of Health. K.Ragunath received educational grants from Erbe and Medtronic. R.Haidry received research infrastructure support from Medtronic, Cook Endoscopy, and Pentax Europe. L.B. Lovat received research infrastructure support from Medtronic. All other authors disclosed no financial relationships relevant to this article.

For peer review only

Abstract

Objectives

Strong recruitment and retention into randomised controlled trials involving invasive therapies is a matter of priority to ensure better achievement of trial aims. The BRIDE (Barrett's Randomized Intervention for Dysplasia by Endoscopy) study investigated the feasibility of undertaking a multi-centre randomised controlled trial comparing Argon Plasma Coagulation and Radiofrequency Ablation, following endoscopic resection, for the management of early Barrett's neoplasia. This paper aims to identify factors influencing patients' participation in the BRIDE study and determine their views regarding acceptability of a potential future trial comparing surgery to endotherapy.

Design

A semi-structured telephone interview study was performed, including both patients who accepted and declined to participate in the BRIDE trial. Interview data was analysed using the constant comparison approach to identify recurring themes.

Setting

Interview participants were recruited from across six UK tertiary centres where the BRIDE trial was conducted.

For peer review only

Participants

We interviewed 18 participants, including eleven participants in the BRIDE trial and seven who declined.

Results

Four themes were identified centred around interviewees' decision to accept or decline participation in the BRIDE trial and a potential future trial comparing endotherapy to surgery: (i) influence of the recruitment process and participant-recruiter relationship, (ii) participants' views of the design and aim of the study (iii) conditional altruism as a determining factor and (iv) participants' perceptions of surgical risks versus less invasive treatments.

Conclusion

We identified four main influences to optimising recruitment and retention to a randomised controlled trial comparing endotherapies in patients with early Barrett's related neoplasia. These findings highlight the importance of qualitative research to inform the design of larger randomised controlled trials.

Keywords

Barrett's oesophagus, interview, endotherapy, oesophageal cancer, qualitative, randomised controlled trial

For peer review only

Strengths and limitations of the study

- This qualitative study synthesises evidence from both patients who accepted to participate and those who rejected participation in a feasibility RCT, allowing a broader understanding of factors influencing participation in RCTs involving endoscopic procedures for the management of Barrett's related early neoplasia and those comparing endoscopy to surgery.
- Participants were recruited from all six participating sites in the BRIDE trial, maximising transferability of results.
- The interviews were conducted by telephone, allowing interviewees to participate from the comfort of their own familiar environments.
- The conduct of interviews by telephone also limited the ability of the interviewer to detect non-visual cues from the participants.
- The study was conducted before the COVID pandemic. Different themes may have emerged from repeating the study after the pandemic.

For peer review only

Background

Randomised controlled trials (RCTs) are widely recognised as the most robust way of determining cause and effect between treatment and outcomes.[1] To ensure statistical power and validity of results, strong recruitment and retention into RCTs is a matter of priority for investigators, clinicians and funders. A review of trials funded by two of the UK's biggest funding agencies (the UK Medical Research Council and the National Institute of Health Research Health Technology Assessment Programme) found that less than a third of trials were able to achieve their recruitment targets.[2] Two Cochrane reviews investigating influences to patient participation in RCTs have identified some effective interventions, such as telephone reminders to non-respondents, use of opt-out instead of opt-in procedures for initiating patient contact and open trial designs, but many such strategies may also pose ethical issues.[3]

Factors affecting the recruitment and retention of participants into research involving endoscopy interventions have not previously been investigated. Yet, there is a need for adequately powered RCTs to determine both safety and effectiveness of such invasive procedures when comparing different types of endotherapies with each other and with more invasive surgical alternatives. One area of gastrointestinal medicine where therapeutic options include both endotherapies and surgery is the management of early oesophageal cancer secondary to Barrett's oesophagus (BO). Barrett's oesophagus is a premalignant condition where the stratified squamous epithelial lining of the distal oesophagus is replaced with a pathological, specialised columnar epithelium. It occurs as a consequence of chronic inflammation from gastroesophageal reflux disease.[4] It may further progress through degrees of dysplasia to adenocarcinoma. In the presence of high grade dysplasia

For peer review only

(HGD), BO carries a high risk of progression[5, 6] which, if diagnosed once symptoms have occurred, has a very poor prognosis with five year survival rates of around 10-15 % in England and Wales.[7] If HGD or intramucosal cancer are identified, macroscopically visible lesions are usually removed by endoscopic resection (ER) but it may leave behind BO where pre-cancerous lesions (dysplasia) can recur.[8]

The aim of the BRIDE (Barrett's Randomised Intervention for Dysplasia by Endoscopy) study was to investigate the feasibility of undertaking a multi-centre RCT comparing two ablative endotherapies used in addition to ER in the management of early Barrett's related neoplasia (HGD or T1 adenocarcinoma): Argon Plasma Coagulation (APC) and Radiofrequency Ablation (RFA), both of which had shown promising results in clinical trials where they were compared for post-ER mucosal ablation to surveillance[9] and sham.[10] This paper describes factors identified from semi-structured interviews of patients approached to participate in BRIDE, influencing their decision to accept or decline to participate and to determine their views regarding acceptability of a potential future trial comparing surgery to endotherapy.

Methods

This qualitative study consisted of semi-structured telephone interviews with a purposive sample of patients contacted for participation in BRIDE. Patients who were contacted to enroll in BRIDE were asked if they would be willing to take part in an interview study to explore views on recruitment to clinical trials in this area. More details on the patient identification process from the six participating English centres has been described previously.[11] They were chosen to represent a broad range of characteristics based on age, sex, centre and decision to participate or not participate in the trial. Participants who were

For peer review only

interested in taking part in the interview study were sent an information sheet by post, with return envelopes for signed consent forms and contact details for the telephone interview.

To explore the feasibility of conducting larger fully powered trials, the interviews focused on views and experiences of being invited to participate in BRIDE and participation in a potential future trial of endotherapy versus surgery. Interviews were conducted by a qualitative researcher in the first year after being approached, and was structured flexibly around an interview topic guide, developed with lay representatives (supp material A).

All tapes were transcribed verbatim and anonymized. Analysis was based on the constant comparative method[12] assisted by QSR N6 software. A coding framework was developed through detailed and repeated inspection of the transcripts, initially to identify textual units of meaning, which were then organized into higher order thematic categories.

The study received ethical approval from the Leicester Central National Research Ethics Service Committee, East Midlands Research Ethics Committee (12/EM/0445).

Patient and Public Involvement

The interview topic guide was developed collaboratively with two members of the public with lived experience of oesophageal cancer as a patient and as a carer.

Results

We interviewed 18 participants: 16 men and two women, aged 47 to 85 from all six treatment centres. Eleven interviewees participated in the BRIDE trial and seven chose not to. Interviews lasted between 26 to 79 minutes. When discussing factors influencing their decisions whether or not to enrol into the BRIDE trial and a potential future trial comparing endotherapy to surgery, participants mentioned a number of considerations, which have

For peer review only

been synthesized into four main themes describing factors intrinsic to: (i) the nature of the trial recruitment process along with the participants' relationship with the trial recruiters and clinical staff, (ii) the participants' perceptions of the design and aim of the study, (iii) participants' motivations for participation balanced against the burden of participation (conditional altruism), and (iv) participants' perceptions of risks of surgery versus endotherapies. These themes are described in more details below:

Nature of recruitment process and role of recruiters

Participants valued the interpersonal skills of the recruiters, irrespective of their grades or professions, placing emphasis on their attitudes and respectful approach. Such attributes translated to a smooth recruitment process to the trial, thereby ensuring that participants did not feel any pressure to participate.

> *"I mean she [the recruiter] was very pleasant …and I think if you've got a disposition like that…it puts you at ease … there's no way that I thought to myself 'I'm pressurised into it' and I fully understood that if ever I wanted to pull out of the study then I can."* (Participant 12)

> *"…it's a very sort of informal situation which I was very comfortable with. I think I'd find it more difficult if I had some very formal doctor. I think the relationship I just feel was being, right from the first I found it very easy to talk to and they were very open in all aspects and they were very inclusive of the patient down there."* (Participant 14)

Participants also placed considerable trust in their clinical team, whom they viewed as the experts in managing their condition. A product of this trust was the belief that, if their

For peer review only

clinical team was discussing the option of enrolling into a particular trial, it had to be beneficial for them. Such trust lessened the weight participants placed on written information provided to them as part of the recruitment process, and facilitated their decision-making.

> *"…if he [the consultant] said that he reckoned he could improve the situation, then I was quite happy at just letting him get on with it."* (Participant 15)

> *"I'd already had some treatments from [Doctor A], and I'd got every confidence in him, so when he came along and said, 'We want to do this and we want to do that', I'd every confidence in him."* (Participant 13)

Our findings illustrate the importance of adequate planning when approaching patients for recruitment into trials. On two occasions, participants received information about the trial before being told that they had early cancer, creating significant distress leading to one of the participants declining to be part of the trial. Approaches seen as unconventional by patients, such as being called to a different room or their clinician asking for a nurse or another clinician to be present, caused concern to patients.

> *"The first time I heard about [the trial] was when I received information through the post… But I hadn't even had my results back from the hospital. Nobody had explained to me what Barrett's Oesophagus was…so to then receive the package, because it was quite a substantial package with consent forms and everything…I wasn't contacted after I had my results or sat down with anybody, I was contacted beforehand and that was a bit alarming…Not just for myself …it was quite upsetting for my wife and my daughter."* (Participant 6)

For peer review only

"Before we went to see the surgeon, he [the researcher] wrote a letter to us saying that 'oh you've been diagnosed with this cancer' and he wanted to study, well we didn't know about it… they said 'oh, [Dr B] wants to see you.. and your wife'… we went to see him…and the wife says to him 'OK, if it's bad news, give us the bad news', 'oh no, no', because he'd called in another consultant as well, he called in another consultant and we were wondering… why is he calling another consultant [trial investigator] in, I thought that this meant bad…" (Participant 18)

Participants' perceptions of the study

Participants' understanding of the purpose of the trial varied widely. Participants conflated the aims of the *feasibility* study which they were enrolling in (which was to investigate whether it was possible to conduct a more definitive adequately powered future study) with that of a *definitive* study itself. Those who agreed to participate largely understood that available evidence at the time of the trial showed that both interventions were effective. This belief in clinical equipoise was essential to participants' acceptance for enrolling into the trial and be randomised to one intervention or the other.

"People might be a bit reluctant if they think there's a 50/50 chance of them getting a treatment that's more painful or hard to go through, whereas if both options were pretty much the same, which I believe is so in my case, I've got to have treatment so it's a flip of the coin which I get, I don't really mind." (Participant 9)

"Reading on the Internet what it was about, the two procedures and I just got it in my mind that there were roughly the same treatment…" (Participant 12)

For peer review only

After making their own research into the two interventions, one interviewee did not believe that both treatments were equal, and thereby declined to be part of the trial, choosing instead to receive, what they believed, was the more effective treatment.

> *"I'd done some research…my wife had looked into it as well on the treatment and I couldn't be guaranteed that I'd be given Halo [RFA] treatment. The Halo treatment was a little bit more successful than one of the other treatments that was being offered. So, I then withdrew my consent at the time…"*(Participant 6)

The study design was not always understood by participants, with some believing that their treatment modality was individualised to their needs as opposed to being chosen at random. Such therapeutic misconceptions[13] demonstrated that participants did not always understand the implication of their decisions to enrol in the study and impinged on participants' autonomous decision-making and their ability to give informed consent.

> *"No, he [the clinician] had decided that the gas* [APC] *… would be the best for me… I was quite happy to go along with whatever he suggested."* (Participant 15)

Alarmingly, one participant who had been recruited to take part in the study, did not appear to have any idea what the study was about.

> *"What was the study about, did you understand what was going to go on? [to someone who appeared to be the participant's partner]. … I can't answer your question, I don't know."* (Participant 7)

The implication of non-participation in the trial was also not uniformly understood among participants. Two interviewees believed that active treatment would only be offered to them if they agreed to take part in the trial.

For peer review only

“I don't think [the doctor] really had a choice. It wasn't a no choice it’s a yes go forward because all the choice was just let it carry on and let it take your life isn't it.” (Participant 2)

“I was just approached to say that there were several possible alternative courses of treatment and one of them might involve the BRIDE [trial] effectively. So, I was asked was I willing to consider any of those and I said ‘well, of course, because I want to get better, all being well.” (Participant 17)

Conditional altruism

When discussing reasons for enrolling in the trial, interviewees widely reported being motivated by the need to help others and contribute to medical advancement.

“As I say, it was simply if it helps somebody in the future, good. ’Cause I’m sure somebody [would have] done exactly the same for the treatment I just had.” (Participant 4)

“A letter came through later on and we just thought that we’d help because the National Health, they did a fantastic job on me and basically they saved my life.” (Participant 18)

Such descriptions of altruism and reciprocity were not always unconditional. They were frequently combined with an expectation that participants would also benefit by enrolling in the trial or at least be no worse off. Taking part in the trial was viewed by interviewees as a means of receiving better care by having access to additional information, support and monitoring, which may not be available through standard care.

For peer review only

"… I thought maybe I would get monitored more that was my main reason: I really did think that I would be monitored more.." (Participant 8)

"…I work on the principle that hopefully it will help me and whatever findings come out at the end of the trial hopefully it will help somebody else down the line…" (Participant 11)

"That's right and being on the study, I think you get far better treatment as well." (Participant 16)

As well as considering personal and altruistic motives for participating in the trial, interviewees also gave significance to the amount of personal inconvenience that participating in the trial might entail. Practical concerns included travel, inconvenience to themselves or family, health and financial considerations. Such considerations were largely dependent on the individual's personal circumstances at the time of the approach.

"The main trouble with this study is that it's in [Place 2] and I live in [Place 3], so it's a 40-odd mile trip down. And obviously, having had anaesthetic, I can't drive back. And so one of my daughters had taken me down." (Participant 13)

"The trouble was at the time my late husband was very ill and so it was a question that you know, it was the time factor you know, that I was, I mean OK I wanted to keep myself going for him but also I had to look after him so I couldn't sort of give my full attention to it." (Participant 11)

Risks of surgery

Taking part in the trial was also subject to there being no significant extra treatment or risk to themselves in taking part. Such reflections on risk were fuelled by the fact that both

For peer review only

interventions were endotherapies, considered to have similar risk profiles, unlike more invasive surgery.

> *"…I don't think I had anything to worry about, it [either endotherapy] wasn't going to hurt me or set me back or done any damage to me, it wasn't a risk that way really…they're trying to save you the trauma of a big operation."* (Participant 18)

> *"I think as I've already said, as long as I haven't got to have any extra treatment and it's not going to be much trouble."* (Participant 9)

When presented with information about a potential future trial comparing surgery to endotherapy for the management of the same condition, interviewees recognised the differences in the two interventions much more readily. They were not seen as broadly equivalent. Surgery, for many, was seen as riskier and involved additional suffering alongside a longer recovery time. Therefore, participants rejected the notion of clinical equipoise in such a potential future trial.

> *"I think really if it was going to be a flip of a coin, I think that's no way to treat a patient is it."* (Participant 7)

> *"…anything that can be done with an endoscopy must be better than cutting people open. Would have to be at a much greater, later stage and need for people to be opened up."* (Participant 8)

In order to participate in a trial which might involve surgery as intervention when compared to endotherapy, participants felt they would require more information on efficacy and safety. Ironically both of these determinants would not be known before the trial, since a

For peer review only

trial comparing surgery with endotherapy in the management of early Barrett's related oesophageal cancer would seek to address these exact answers.

> *"Well, if they [researchers] said either is possible, they [participants]'d want to know which one is the most successful."* (Participant 4)

> *"It depends why they [the researchers] want to do it and what are the advantages of having a surgical procedure as opposed to an endoscopy... But I wouldn't say I wouldn't have it, I would want to know a hell of a lot more about it and what the pros and cons are."* (Participant 1)

Discussion

This study highlights important patient-centred considerations when designing and conducting trials involving endotherapies for early oesophageal cancer. Our findings demonstrate the interactions of recruiters with potential participants before recruitment played a key role in influencing recruitment. Participants valued the respectful and pleasant nature of these interactions, particularly when they were not placed under any duress to participate. Recruitment was facilitated by the dual role played by recruiters who were also often the participants' trusted clinicians. This finding mirrors the results of other interview studies of trial recruiters.[14, 15]

Being both a patient's clinician and a trial researcher can be associated with certain emotional conflicts for the clinical-researcher, which need to be acknowledged.[14, 16] For instance, in an interview study, Donovan et al. highlight challenges expressed by clinicians recruiting into trials around their own treatment preferences,[14] based on their personal opinions. Equally, patient's trust in their clinicians should not be abused to facilitate

For peer review only

recruitment. Recruiting clinicians have a duty to be honest and convey both the genuine uncertainty between the safety and effectiveness of the different interventions in the trial (i.e. the clinical equipoise) and be clear on the purpose of feasibility trials, which are designed to inform the design of future more definitive trials. We found both concepts to be poorly understood by certain participants, highlighting potential gaps in the explanation conveyed to them. The literature is also lacking on how recruiters describe and operationalise the concept of equipoise during RCT recruitment.[14, 17]

Our findings highlight the importance of judiciously timed discussions regarding enrolment into studies, in particular where breaking bad news may be involved. We identified instances when patients were contacted to enrol in research before being told of the diagnosis and management options. We suspect that this situation arose because potential participants were identified through Multi-Disciplinary-Team (MDTs) meetings by researchers who were not part of their usual clinical teams. While trial recommendation through MDTs have previously been shown to increase recruitment rates,[18] our findings suggest that researchers should not fall into the trap of "recruitment enthusiasm" and ensure that patients are aware of their diagnoses before contact is made. Unexpected deviations from usual outpatient practices when recruiting patients into trials (e.g. having two consultants seeing a patient) also created undue distress.

While altruism did play a factor in influencing recruitment, we found that participants also considered the degree of inconvenience trial participation would entail. McCann et al. previously coined the term "conditional altruism" to describe participants' willingness to participate in trials to contribute to a greater good so long as they reap some personal benefit from the trial or at least not get harmed.[19] We find that such conditional altruism

For peer review only

also entailed an expectation that participation would not be accompanied by personal inconvenience. This finding is particularly relevant for interventions delivered in tertiary centres, as is recommended for the endoscopic management of patients with Barrett's related neoplasia,[20] and which may involve participants' time and financial sacrifice, as was the case for the BRIDE trial.

Our findings would suggest that surgery would not be acceptable for participants invited to participate in a trial comparing surgery to endotherapy for early oesophageal cancer unless there was a potential advantage to the more invasive intervention (i.e. surgery) such as higher likelihood of cure, counterbalancing the downsides of increased likelihood of complications.[21-24] A recent quality of life survey comparing patients with neoplastic Barrett's oesophagus who had undergone endotherapy to those who had oesophagectomy support this view with the latter group suffering from more long-term symptomatic burden.[25] The issue of recruitment into trials involving surgical interventions is not new, with multiple previous reviews highlighting numerous barriers to such RCTs: the irreversible nature of surgery, strong patient treatment preferences, surgeons' personal opinions and concerns around the idea of randomization.[22-24] We suggest that surgery is only considered an option when compared to endotherapy when there is an established evidence base for the surgical intervention such as high likelihood of cure, counterbalancing the downsides of increased likelihood of complications. For instance, for early oesophageal cancer, there are certain histological prognostic factors which increase the likelihood of lymphatic spread (such as submucosal invasion to more than 500 microns, or lympho-vascular invasion or poorly differentiated tumours).[26, 27] In such cases, surgery would offer a higher likelihood of cure since local lymph nodes are also removed with the oesophageal resection.[28]

For peer review only

Based on our findings, we suggest some practical considerations to maximising recruitment into future RCTs comparing different endotherapies for the management of patients with early oesophageal cancer. These recommendations may be generally applicable to other RCTs involving invasive interventions.

First, there is a need for better coordination between clinical and research teams when contacting potential participants for enrolment. If participants are identified through MDTs, they should only be contacted for participation after being made aware of their diagnoses and treatment options by their clinicians. Clearly, such coordination is made smoother if their own clinician is the trial researcher.

Second, trial recruiters need to acknowledge difficulties in explaining complex concepts such as equipoise and randomisation to patients. Previous trials have used peer feedback to recruiters to improve their own communication and enhanced patient written communication including descriptions of complex trial concepts and interventions in lay-man's terms.[29]

Third, monetary incentives to, at the very least, cover subsistence expenses need to be considered.[30] Such incentives are particularly necessary when patients and carers are expected to cover long distances for treatment as part of a trial.

Fourth, we suggest that multi-centre trials need to include opportunities for group feedback during the conduct of a trial to allow researchers to share good practice and discuss challenges.

This study is limited by the small sample although the qualitative methodology used means that the findings are still valid given that sample size in such research is guided by

For peer review only

theoretical saturation.[31] Nonetheless, a bigger sample may have led to more themes, as would have inclusion of trial researchers among the interviewees. The study was also conducted before the COVID pandemic. Given the effect of the COVID pandemic on patients' lifestyles and access to care, other themes may have been generated from repeating the study during or after the pandemic.

In conclusion, recruitment and retention of participants into RCTs involving endotherapies is not straightforward. We have demonstrated the value of conducting qualitative research as part of feasibility trials to inform the design of larger RCTs. We highlight both opportunities and challenges in maximising recruitment to future trials comparing endotherapies and endotherapy versus surgery for the management of patients with early oesophageal cancer.

Based on our findings, we have made some practical recommendations to optimising recruitment and retention in RCTs involving invasive treatments (both endotherapy and/or surgery) for the management of early BO-related neoplasia. These recommendations may be generally applicable to other RCTs involving invasive interventions. Future research should aim to evaluate the views of researchers and clinicians on factors influencing participation in trials comparing different endotherapies for the management of BO-related early oesophageal cancer and the effect of the suggested recommendations on recruitment.

Ethics

The study received ethical approval from the Leicester Central National Research Ethics Service Committee, East Midlands Research Ethics Committee (12/EM/0445).

For peer review only

References

(1) Altman DG. Better reporting of randomised controlled trials: the CONSORT statement. *BMJ (Clinical research ed.)* 1996 September 07;**313**(7057):570-571.

(2) McDonald AM, Knight RC, Campbell MK*, et al*. What influences recruitment to randomised controlled trials? A review of trials funded by two UK funding agencies. *Trials* 2006;**7**(1):1-8.

(3) Treweek S, Lockhart P, Pitkethly M*, et al*. Methods to improve recruitment to randomised controlled trials: Cochrane systematic review and meta-analysis. *BMJ open* 2013;**3**(2).

(4) Spechler SJ, Goyal RK. The columnar-lined esophagus, intestinal metaplasia, and Norman Barrett. *Gastroenterology* 1996;**110**(2):614-621.

(5) Bhat S, Coleman HG, Yousef F*, et al*. Risk of malignant progression in Barrett's esophagus patients: results from a large population-based study. *Journal of the National Cancer Institute* 2011;**103**(13):1049-1057.

(6) Skinner DB, Walther BC, Riddell RH*, et al*. Barrett's esophagus. Comparison of benign and malignant cases. *Annals of Surgery* 1983;**198**(4):554.

(7) Cancer Research UK. Oesophageal cancer incidence statistics. 2020; Available at: https://www.cancerresearchuk.org/health-professional/cancer-statistics/statistics-by-cancer-type/oesophageal-cancer/incidence#heading-Three. Accessed 04/03/, 2021.

For peer review only

(8) Fitzgerald RC, di Pietro M, Ragunath Kea. Guidelines on the Diagnosis and Management of Barrett's Oesophagus - An Update. LGD: surveillance or ablation? 2015; Available at: http://www.bsg.org.uk/images/stories/docs/clinical/guidelines/oesophageal/bsg_barretts_addendum_15.pdf. Accessed Feb/01, 2017.

(9) Manner H, Rabenstein T, Pech O*, et al*. Ablation of residual Barrett's epithelium after endoscopic resection: a randomised long-term follow-up study of argon plasma coagulation vs. surveillance (APE study). *Endoscopy* 2014;**46**(01):6-12.

(10) Shaheen NJ, Sharma P, Overholt BF*, et al*. Radiofrequency ablation in Barrett's esophagus with dysplasia. *New England Journal of Medicine* 2009;**360**(22):2277-2288.

(11) Peerally MF, Bhandari P, Ragunath K*, et al*. Radiofrequency ablation compared with argon plasma coagulation after endoscopic resection of high-grade dysplasia or stage T1 adenocarcinoma in Barrett's esophagus: a randomised pilot study (BRIDE). *Gastrointestinal endoscopy* 2019;**89**(4):680-689.

(12) Charmaz K, Belgrave LL. Grounded theory. *The Blackwell encyclopedia of sociology* 2007;.

(13) Appelbaum PS, Roth LH, Lidz C. The therapeutic misconception: informed consent in psychiatric research. *International journal of law and psychiatry* 1982;**5**(3-4):319-329.

(14) Donovan JL, Paramasivan S, de Salis I*, et al*. Clear obstacles and hidden challenges: understanding recruiter perspectives in six pragmatic randomised controlled trials. *Trials* 2014;**15**(1):5.

For peer review only

(15) Donovan JL, de Salis I, Toerien M, *et al*. The intellectual challenges and emotional consequences of equipoise contributed to the fragility of recruitment in six randomised controlled trials. *Journal of clinical epidemiology* 2014;**67**(8):912-920.

(16) Elliott D, Husbands S, Hamdy FC, *et al*. Understanding and improving recruitment to randomised controlled trials: qualitative research approaches. *European urology* 2017;**72**(5):789-798.

(17) Garcia J, Elbourne D, Snowdon C. Equipoise: a case study of the views of clinicians involved in two neonatal trials. *Clinical trials* 2004;**1**(2):170-178.

(18) McNair A, Choh C, Metcalfe C, *et al*. Maximising recruitment into randomised controlled trials: the role of multidisciplinary cancer teams. *European journal of cancer* 2008;**44**(17):2623-2626.

(19) McCann SK, Campbell MK, Entwistle VA. Reasons for participating in randomised controlled trials: conditional altruism and considerations for self. *Trials* 2010;**11**(1):31.

(20) Alzoubaidi D, Ragunath K, Wani S, *et al*. Quality indicators for Barrett's endotherapy (QBET): UK consensus statements for patients undergoing endoscopic therapy for Barrett's neoplasia. *Frontline gastroenterology* 2019 August 14;**11**(4):259-271.

(21) Rutegård M, Lagergren P, Rouvelas I, *et al*. Surgical complications and long-term survival after esophagectomy for cancer in a nationwide Swedish cohort study. *European Journal of Surgical Oncology (EJSO)* 2012;**38**(7):555-561.

For peer review only

(22) McLeod RS. Issues in surgical randomised controlled trials. *World journal of surgery* 1999;**23**(12):1210-1214.

(23) Lilford R, Braunholtz D, Harris J*, et al*. Trials in surgery. *British journal of surgery* 2004;**91**(1):6-16.

(24) Abraham NS, Young JM, Solomon MJ. A systematic review of reasons for nonentry of eligible patients into surgical randomised controlled trials. *Surgery* 2006;**139**(4):469-483.

(25) Reddy CA, Tavakkoli A, Chen VL*, et al*. Long-Term Quality of Life Following Endoscopic Therapy Compared to Esophagectomy for Neoplastic Barrett's Esophagus. *Digestive diseases and sciences* 2021;**66**(5):1580-1587.

(26) Herrero LA, Pouw RE, Van Vilsteren F*, et al*. Risk of lymph node metastasis associated with deeper invasion by early adenocarcinoma of the esophagus and cardia: study based on endoscopic resection specimens. *Endoscopy* 2010;**42**(12):1030-1036.

(27) Lorenz D, Origer J, Pauthner M*, et al*. Prognostic risk factors of early esophageal adenocarcinomas. *Annals of Surgery* 2014;**259**(3):469-476.

(28) Mariette C, Dahan L, Mornex F*, et al*. Surgery alone versus chemoradiotherapy followed by surgery for stage I and II esophageal cancer: final analysis of randomised controlled phase III trial FFCD 9901. *J Clin Oncol* 2014;**32**(23):2416-2422.

(29) Rooshenas L, Scott LJ, Blazeby JM*, et al*. The QuinteT Recruitment Intervention supported five randomised trials to recruit to target: a mixed-methods evaluation. *Journal of clinical epidemiology* 2019;**106**:108-120.

For peer review only

(30) Caldwell PH, Hamilton S, Tan A*, et al*. Strategies for increasing recruitment to randomised controlled trials: systematic review. *PLoS Med* 2010;**7**(11):e1000368.

(31) Bryman A. *Social Research Methods.* New York: Oxford University Press; 2012.

For peer review only

Data availability statement

Data are available upon reasonable request.

Acknowledgements

We would like to thank our two expert patient advisers for their contribution in the design of the study.

Author statement

MFP wrote the initial manuscript and edited subsequent versions following feedback from all authors. CJ performed the analysis of the data and provided a data summary to all authors. CJ, KR, HB, CS, RH, LBL, HS critically revised and edited all versions. JdC reviewed all versions of the manuscript and edited the final version of the manuscript. All authors approved the final manuscript version being submitted for publication.

Grant support

This article presents independent research funded by the National Institute for Health Research (NIHR) under its Research for Patient Benefit Programme (grant no. PB-PG-0711-25066).

Disclosure

The views expressed are those of the authors and not necessarily those of the NHS, the NIHR, or the Department of Health. K.Ragunath received educational grants from Erbe and Medtronic. R.Haidry received research infrastructure support from Medtronic, Cook

For peer review only

Endoscopy, and Pentax Europe. L.B. Lovat received research infrastructure support from Medtronic. All other authors disclosed no financial relationships relevant to this article.

For peer review only

Supplementary material A – Interview Topic Guide

This guide is designed to be used flexibly to encourage patients to talk about what matters to them. If the patient brings up an issue not covered here, this will be actively explored. The content is led by the patient, the interviewer's role is to prompt in a way that helps to address the research focus, which is to identify and understand the nature of barriers and enablers to participation in studies of this kind.

The layout of the topic guide may also be amended to suit the individual doing the interviews.

Preliminaries, not necessarily in this order, to be determined by interviewer:

Check that timing of phone call is still convenient.

Introduce interviewer.

Go through information sheet; give opportunity to ask questions, especially confidentiality, anonymity issues, audio recording and transcribing (why it is important).

Confidentiality – what you say will not get back to the center treating you; interviews will be analysed all together, all identifying details removed, and themes summarised. For instance, we might find that several people mention a particular thing – for instance there might be an issue about not feeling rushed – and we will report that; but we would not mention your name or the names of doctors or nurses.

The only situation in which I would break confidentiality is if I heard that someone's life was in danger – this is most unlikely in an interview like this.

For peer review only

Talk through nature of interview – i.e. I am interested in your views so there are no wrong or right answers. I have a list of topics, which is here just to start off the conversation, but what we are really interested in is what matters to you.

Can stop at any time, just ask;

Can say you'd prefer to move on to the next question if there is something that it's not convenient to talk about at the moment

We can take as long as you like, or stop at a particular time – do you have a particular time you need to finish?

Any questions?

We are interested in how people feel about taking part in studies of the sort of treatment you have been having, so we are talking to you because you were asked to take part in a study to do with the treatment of your gullet. Can I check first, is that right?

DECIDING

Some people make up their minds really quickly about taking part in research. Others prefer to weigh things up for longer. How tricky was it for you to decide?

What sorts of things led to your decision?

Prompts

WHO AND HOW recruited

Did it matter WHO asked you, or would you have just said yes/no anyway?

For peer review only

Did it matter HOW it was explained, how much information did you want, what sort of information (explore role of written information, opportunities to talk and ask questions), or would you have said yes anyway …

SHARED OR SOLE DECISION

Many people see this sort of thing as solely their own decision, others make a decision but also like to discuss it with family or friends, how did you feel? (did you talk to partner/family/others about it – tell me a bit more about that)

How did partner/family/others feel about you taking part, did that make a difference to you?

If applicable – how much information did you husband/wife/family want? Who explained it to them? How well did this work out do you think?

PERCEPTION OF AIM AND STRUCTURE OF STUDY

I wondered how it feels to be a patient – were there parts of the study you understood better than others? Did you feel as if you understood what the study aims to do? Can you tell me about that? Were there some things that are harder to understand? (For you, or maybe even if it was OK for you, were there parts that might be hard for other patients). How does it feel to be in the study? How do you think you'd feel if you'd decided against it?

Was there anything that made it easy for you to decide?

Was there anything that made it difficult to decide?

For peer review only

LOOKING BACK:

Looking back, do you feel positive about deciding (not) to take part, or are there some things you are less positive about? (or if you prefer not to look back that's fine, just say so) – What has your treatment been like? How did it feel? Do you know what the treatment was? How was your treatment decided?

If participated: We hope that this didn't happen, but did you at any point feel like dropping out of the study? Tell me about that (what meant that you carried on, how difficult was it, did someone or something make a difference, was there anything someone could have done that would have made that easier etc.)

IF PARTICIPATED: VIEWS ON TAKING PART:

In your view, what have been the good things and the less good things about taking part in the study? Take them in whatever order suits you.

Keep prompting until covered

VIEWS ON TREATMENT:

And in a similar vein, (if not already covered), what have been the good points and the bad points about the treatment you have been receiving? Feel free to mention anything even if it seems minor, because it's important to know what it is like for patients – you are the only expert at being on the receiving end.

For peer review only

Are there any things that you think would help with future studies –?

Things that would help people to decide whether or not to take part

Things that would help people to carry on /not 'drop out',

FUTURE TRIALS:

Now I'd like to ask a few final questions. The treatment centers involved in this study would like to do another piece of research, comparing endoscopic treatment to surgery. We are NOT asking you to take part in this. The reason I am asking you about it is to get your advice because you have some experience of what it's like to be in a study.

Imagine patients were told that there was a need to compare a surgical treatment with an endoscopic treatment because although both seem roughly equivalent, in terms of success, we don't know all their advantages and disadvantages. Taking part would not benefit the patients themselves, but it would provide information that would help in treating people in the future.

How do you think people would feel to be asked whether they were be willing to receive either surgery, or endoscopic treatment, at random (i.e. they would have a fifty fifty chance of each treatment – like deciding by tossing a coin)?

What sorts of things would they want to know before they decided? (explore)

What sorts of things might put them off taking part? (explore)

What sorts of things might mean they were likely to take part? (explore)

For peer review only

Any general messages for people who design and run studies? (i.e. things that would make life easier for those being asked to take part, things that some centers do really well when it comes to research, that other doctors could learn from – or the opposite)

Anything else you want to say about taking part in this type of research? For instance is there anything that makes research into this condition (gullet-related) different to research in other conditions? Tell me a bit about it.

Last - What do you think researchers should be trying to achieve in the future? What should they be looking at, to help with this condition?

Is there anything else you want to say, or to ask me? I cannot answer questions about your particular treatment but I can pass on queries.

Give contact details in case participant wishes to add or retract anything

Thanks

For peer review only

Standards for Reporting Qualitative Research (SRQR)*

http://www.equator-network.org/reporting-guidelines/srqr/

Page/line no(s).

Title and abstract

**Title** - Concise description of the nature and topic of the study Identifying the study as qualitative or indicating the approach (e.g., ethnography, grounded theory) or data collection methods (e.g., interview, focus group) is recommended	Page 1 Line 2-3
**Abstract** - Summary of key elements of the study using the abstract format of the intended publication; typically includes background, purpose, methods, results, and conclusions	Page 5 Lines 1-17; Page 6 Lines 1-15

Introduction

**Problem formulation** - Description and significance of the problem/phenomenon studied; review of relevant theory and empirical work; problem statement	Start of page 8 to Page 9 Line 4
**Purpose or research question** - Purpose of the study and specific objectives or questions	Page 9, lines 5-13

Methods

**Qualitative approach and research paradigm** - Qualitative approach (e.g., ethnography, grounded theory, case study, phenomenology, narrative research) and guiding theory if appropriate; identifying the research paradigm (e.g., postpositivist, constructivist/ interpretivist) is also recommended; rationale**	Page 9, line 14-16
**Researcher characteristics and reflexivity** - Researchers' characteristics that may influence the research, including personal attributes, qualifications/experience, relationship with participants, assumptions, and/or presuppositions; potential or actual interaction between researchers' characteristics and the research questions, approach, methods, results, and/or transferability	Page 10, line 3-5
**Context** - Setting/site and salient contextual factors; rationale**	Page 9, line 16-21
**Sampling strategy** - How and why research participants, documents, or events were selected; criteria for deciding when no further sampling was necessary (e.g., sampling saturation); rationale**	Page 9, lines 20-21
**Ethical issues pertaining to human subjects** - Documentation of approval by an appropriate ethics review board and participant consent, or explanation for lack thereof; other confidentiality and data security issues	Page 10, lines 10-11
**Data collection methods** - Types of data collected; details of data collection procedures including (as appropriate) start and stop dates of data collection and analysis, iterative process, triangulation of sources/methods, and modification of procedures in response to evolving study findings; rationale**	Page 10, line 3-9

For peer review only

For peer review only - http://bmjopen.bmj.com/site/about/guidelines.xhtml

**Data collection instruments and technologies** - Description of instruments (e.g., interview guides, questionnaires) and devices (e.g., audio recorders) used for data collection; if/how the instrument(s) changed over the course of the study	Page 10, line 3-9
**Units of study** - Number and relevant characteristics of participants, documents, or events included in the study; level of participation (could be reported in results)	Page 10, lines 13
**Data processing** - Methods for processing data prior to and during analysis, including transcription, data entry, data management and security, verification of data integrity, data coding, and anonymization/de-identification of excerpts	Page 10, lines 6-9
**Data analysis** - Process by which inferences, themes, etc., were identified and developed, including the researchers involved in data analysis; usually references a specific paradigm or approach; rationale**	Page 10, lines 6-9
**Techniques to enhance trustworthiness** - Techniques to enhance trustworthiness and credibility of data analysis (e.g., member checking, audit trail, triangulation); rationale**	Page 10, lines 6-9

Results/findings

**Synthesis and interpretation** - Main findings (e.g., interpretations, inferences, and themes); might include development of a theory or model, or integration with prior research or theory	Page 10, lines 12-23, pages 11-17
**Links to empirical data** - Evidence (e.g., quotes, field notes, text excerpts, photographs) to substantiate analytic findings	pages 11-17

Discussion

**Integration with prior work, implications, transferability, and contribution(s) to the field -** Short summary of main findings; explanation of how findings and conclusions connect to, support, elaborate on, or challenge conclusions of earlier scholarship; discussion of scope of application/generalizability; identification of unique contribution(s) to scholarship in a discipline or field	Start of page 18 to page 21, line 11
**Limitations** - Trustworthiness and limitations of findings	Page 21, lines 12-18

Other

**Conflicts of interest** - Potential sources of influence or perceived influence on study conduct and conclusions; how these were managed	Page 27, line 16-20
**Funding** - Sources of funding and other support; role of funders in data collection, interpretation, and reporting	Page 27, line 11-14

*The authors created the SRQR by searching the literature to identify guidelines, reporting standards, and critical appraisal criteria for qualitative research; reviewing the reference lists of retrieved sources; and contacting experts to gain feedback. The SRQR aims to improve the transparency of all aspects of qualitative research by providing clear standards for reporting qualitative research.

For peer review only - http://bmjopen.bmj.com/site/about/guidelines.xhtml

**The rationale should briefly discuss the justification for choosing that theory, approach, method, or technique rather than other options available, the assumptions and limitations implicit in those choices, and how those choices influence study conclusions and transferability. As appropriate, the rationale for several items might be discussed together.

Reference:
O'Brien BC, Harris IB, Beckman TJ, Reed DA, Cook DA. **Standards for reporting qualitative research: a synthesis of recommendations.** *Academic Medicine*, Vol. 89, No. 9 / Sept 2014 DOI: 10.1097/ACM.0000000000000388

For peer review only

For peer review only - http://bmjopen.bmj.com/site/about/guidelines.xhtml

BMJ Open

Factors influencing participation in randomised clinical trials among patients with Barrett's early neoplasia: A multi-centre interview study

Journal:	*BMJ Open*
Manuscript ID	bmjopen-2022-064117.R1
Article Type:	Original research
Date Submitted by the Author:	18-Nov-2022
Complete List of Authors:	Peerally, Mohammad; University of Leicester,<br>Jackson, Clare; University of Nottingham Faculty of Medicine and Health Sciences<br>Bhandari, P; Queen Alexandra Hospital, Portsmouth<br>Ragunath, Krish; Curtin University,<br>Barr, Hugh; Gloucestershire Royal Hospital<br>Stokes, Clive; Gloucestershire Royal Hospital<br>Haidry, Rehan; University College Hospital<br>Lovat, Laurence; University College London, Division of Surgery and Interventional Science<br>Smart, Howard; Royal Liverpool Hospital, Department of Gastroenterology<br>De Caestecker, John; The University of Leicester; University Hospitals of Leicester NHS Trust
**Primary Subject Heading**:	Gastroenterology and hepatology
Secondary Subject Heading:	Qualitative research, Gastroenterology and hepatology
Keywords:	Endoscopy < GASTROENTEROLOGY, Oesophageal disease < GASTROENTEROLOGY, Adult gastroenterology < GASTROENTEROLOGY

SCHOLARONE™ Manuscripts

I, the Submitting Author has the right to grant and does grant on behalf of all authors of the Work (as defined in the below author licence), an exclusive licence and/or a non-exclusive licence for contributions from authors who are: i) UK Crown employees; ii) where BMJ has agreed a CC-BY licence shall apply, and/or iii) in accordance with the terms applicable for US Federal Government officers or employees acting as part of their official duties; on a worldwide, perpetual, irrevocable, royalty-free basis to BMJ Publishing Group Ltd ("BMJ") its licensees and where the relevant Journal is co-owned by BMJ to the co-owners of the Journal, to publish the Work in this journal and any other BMJ products and to exploit all rights, as set out in our licence.

The Submitting Author accepts and understands that any supply made under these terms is made by BMJ to the Submitting Author unless you are acting as an employee on behalf of your employer or a postgraduate student of an affiliated institution which is paying any applicable article publishing charge ("APC") for Open Access articles. Where the Submitting Author wishes to make the Work available on an Open Access basis (and intends to pay the relevant APC), the terms of reuse of such Open Access shall be governed by a Creative Commons licence – details of these licences and which Creative Commons licence will apply to this Work are set out in our licence referred to above.

Other than as permitted in any relevant BMJ Author's Self Archiving Policies, I confirm this Work has not been accepted for publication elsewhere, is not being considered for publication elsewhere and does not duplicate material already published. I confirm all authors consent to publication of this Work and authorise the granting of this licence.

For peer review only

Factors influencing participation in randomised clinical trials among patients with Barrett's early neoplasia: A multi-centre interview study

Authors

Mohammad Farhad Peerally PhD[1,2] , Clare Jackson MSc[3], Pradeep Bhandari MD[4], Krish Ragunath MD[5], Hugh Barr MD[6], Clive Stokes MD[6], Rehan Haidry MD[7], Laurence B Lovat PhD[7,8], Howard Smart MD[9], John S de Caestecker MD[10,11]

Author emails:

mfp6@le.ac.uk (corresponding author)

Clare.Jackson@nottingham.ac.uk

pradeep.bhandari@porthosp.nhs.uk

krish.ragunath@curtin.edu.au

For peer review only - http://bmjopen.bmj.com/site/about/guidelines.xhtml

hugh.barr@glos.nhs.uk

clivestokes@nhs.net

rehan.haidry@nhs.net>

l.lovat@ucl.ac.uk

howard.smart@rlbuht.nhs.uk

john.decaestecker@uhl-tr.nhs.uk

Affiliations

1. Digestive Diseases Unit, Kettering General Hospital, Rothwell Rd, Kettering NN16 8UZ, UK.
2. SAPPHIRE, Department of Health Sciences, College of Life Sciences, George Davies Centre, University of Leicester, University Road, Leicester, LE1 7HA, UK.
3. School of Medicine, University of Nottingham, Queen's Medical Centre, Nottingham, NG7 2UH, UK.
4. Department of Gastroenterology, Queen Alexandra Hospital, Southwick Hill Road, Portsmouth, PO6 3LY, UK.
5. NIHR Nottingham Biomedical Research Centre, Nottingham University Hospitals NHS Trust and the University of Nottingham, Derby Road, Nottingham, NG7 2UH, UK.
6. Department of Surgery, Gloucester Royal Hospital, Great Western Road, Gloucester, Gloucestershire, GL1 3NN, UK.
7. Department of Gastroenterology, University College Hospital, 235 Euston Road, London, NW1 2BU, UK.

For peer review only

8. Division of Surgery and Interventional Science, University College London, Gower Street, London WC1B 6BT
9. Department of Gastroenterology, Royal Liverpool Hospital, Prescot St, Liverpool L7 8XP, UK.
10. Leicester Cancer Research Centre, College of Life Sciences, The University of Leicester, University Road, Leicester, LE1 7RH, UK.
11. Digestive Diseases Centre, University Hospitals of Leicester NHS Trust, Infirmary close, Leicester, LE1 5WW, UK.

Corresponding author

Dr Mohammad Farhad Peerally (mfp6@le.ac.uk)

Room 4.33, George Davies Centre, Department of Health Sciences, College of Life Sciences, George Davies Centre, University of Leicester, University Road, Leicester, LE1 7HA, UK.

Phone: 07977764607

Running head

Influencers to participation in endoscopy RCT

Word count

4269 words

Grant support

This article presents independent research funded by the National Institute for Health Research (NIHR) under its Research for Patient Benefit Programme (grant no. PB-PG-0711-25066).

For peer review only

Trial registration

ClinicalTrials.gov Identifier: NCT01733719

Disclosure

The views expressed are those of the authors and not necessarily those of the NHS, the NIHR, or the Department of Health. K.Ragunath received educational grants from Erbe and Medtronic. R.Haidry received research infrastructure support from Medtronic, Cook Endoscopy, and Pentax Europe. L.B. Lovat received research infrastructure support from Medtronic. All other authors disclosed no financial relationships relevant to this article.

For peer review only

Abstract

Objectives

Strong recruitment and retention into randomised controlled trials involving invasive therapies is a matter of priority to ensure better achievement of trial aims. The BRIDE (Barrett's Randomized Intervention for Dysplasia by Endoscopy) study investigated the feasibility of undertaking a multi-centre randomised controlled trial comparing Argon Plasma Coagulation and Radiofrequency Ablation, following endoscopic resection, for the management of early Barrett's neoplasia. This paper aims to identify factors influencing patients' participation in the BRIDE study and determine their views regarding acceptability of a potential future trial comparing surgery to endotherapy.

Design

A semi-structured telephone interview study was performed, including both patients who accepted and declined to participate in the BRIDE trial. Interview data was analysed using the constant comparison approach to identify recurring themes.

Setting

Interview participants were recruited from across six UK tertiary centres where the BRIDE trial was conducted.

For peer review only

Participants

We interviewed 18 participants, including eleven participants in the BRIDE trial and seven who declined.

Results

Four themes were identified centred around interviewees' decision to accept or decline participation in the BRIDE trial and a potential future trial comparing endotherapy to surgery: (i) influence of the recruitment process and participant-recruiter relationship, (ii) participants' views of the design and aim of the study (iii) conditional altruism as a determining factor and (iv) participants' perceptions of surgical risks versus less invasive treatments.

Conclusion

We identified four main influences to optimising recruitment and retention to a randomised controlled trial comparing endotherapies in patients with early Barrett's related neoplasia. These findings highlight the importance of qualitative research to inform the design of larger randomised controlled trials.

Keywords

Barrett's oesophagus, interview, endotherapy, oesophageal cancer, qualitative, randomised controlled trial

For peer review only

Strengths and limitations of the study

- This qualitative study synthesises evidence from both patients who accepted to participate and those who rejected participation in a feasibility RCT, allowing a broader understanding of factors influencing participation in RCTs involving endoscopic procedures for the management of Barrett's related early neoplasia and those comparing endoscopy to surgery.
- Participants were recruited from all six participating sites in the BRIDE trial, maximising transferability of results.
- The interviews were conducted by telephone, allowing interviewees to participate from the comfort of their own familiar environments.
- The conduct of interviews by telephone also limited the ability of the interviewer to detect non-visual cues from the participants.
- The study was conducted before the COVID pandemic. Different themes may have emerged from repeating the study after the pandemic.

For peer review only

Background

Randomised controlled trials (RCTs) are widely recognised as the most robust way of determining cause and effect between treatment and outcomes.[1] To ensure statistical power and validity of results, strong recruitment and retention into RCTs is a matter of priority for investigators, clinicians and funders. A review of trials funded by two of the UK's biggest funding agencies (the UK Medical Research Council and the National Institute of Health Research Health Technology Assessment Programme) found that less than a third of trials achieved their recruitment targets.[2] Two Cochrane reviews investigating influences to patient participation in RCTs have identified some effective interventions, such as telephone reminders to non-respondents, use of opt-out instead of opt-in procedures for initiating patient contact and open trial designs, but many such strategies may also pose ethical issues.[3]

Factors affecting the recruitment and retention of participants into research involving endoscopy interventions have not been previously investigated. Yet, there is a need for adequately powered RCTs to determine both safety and effectiveness of such invasive procedures when comparing different types of endotherapies with each other and with more invasive surgical alternatives. Studies using qualitative methods can investigate issues relating to trial recruitment which may not be captured using quantitative methods, by exploring the experiences of participants and recruiters.[4] One area of gastrointestinal medicine where therapeutic options include both endotherapies and surgery is the management of early oesophageal cancer secondary to Barrett's oesophagus (BO). Barrett's oesophagus is a premalignant condition where the stratified squamous epithelial lining of the distal oesophagus is replaced with a pathological, specialised columnar epithelium. It

For peer review only

occurs as a consequence of chronic inflammation from gastroesophageal reflux disease.[5] It may further progress through degrees of dysplasia to adenocarcinoma.[6] In the presence of high grade dysplasia (HGD), BO carries a high risk of progression[7, 8] which, if diagnosed once symptoms have occurred, has a very poor prognosis with five-year survival rates of around 10-15 % in England and Wales.[9] If HGD or intramucosal cancer are identified, macroscopically visible lesions are usually removed by endoscopic resection (ER) but it may leave behind BO where pre-cancerous lesions (dysplasia) can recur.[10]

The aim of the BRIDE (Barrett's Randomised Intervention for Dysplasia by Endoscopy) study was to investigate the feasibility of undertaking a multi-centre RCT comparing two ablative endotherapies used in addition to ER in the management of early Barrett's related neoplasia (HGD or T1 adenocarcinoma): Argon Plasma Coagulation (APC) and Radiofrequency Ablation (RFA), both of which had shown promising results in clinical trials where they were compared for post-ER mucosal ablation to surveillance[11] and sham.[12] This paper describes factors identified from semi-structured interviews of patients approached to participate in BRIDE, influencing their decision to accept or decline to participate, and to determine their views regarding acceptability of a potential future trial comparing surgery to endotherapy.

Methods

This qualitative study consisted of semi-structured telephone interviews with a purposive sample of patients contacted for participation in BRIDE. The sampling strategy was designed to include a wide range of views and experiences, including those who agreed to be part of the trial and those who did not, from across all six participating English centres. Potential participants were approached for recruitment into the clinical trial and the interview study

For peer review only

when they attended for either their outpatient appointment or their endoscopy, after being identified at a local cancer multi-disciplinary team meeting (MDT). They were all asked whether they would be willing to participate in the interview study, irrespective of their decision to enlist on the trial. Potential participants were given a minimum of 48 hours to consider the information given to them about enrolment into the trial and/or interview study. More details on the patient identification process from the six participating centres has been described previously.[13] They were chosen to represent a broad range of characteristics based on age, sex, centre and decision to participate or not participate in the trial. Participants who were interested in taking part in the interview study were sent an information sheet by post, with return envelopes for signed consent forms and contact details for the telephone interview.

To explore the feasibility of conducting larger fully powered trials, the interviews focused on views and experiences of being invited to participate in BRIDE and participation in a potential future trial of endotherapy versus surgery. Interviews were conducted by an experienced non-clinical qualitative researcher, who was not involved in the recruitment of patients in the trial. They occurred in the first year after patients were approached, and were structured flexibly around an interview topic guide, developed in an inductive manner by the authors, and informed by discussions with lay representatives (supp material A). No further participants were recruited after achieving theoretical saturation.[14]

All tapes were transcribed verbatim and anonymized. Analysis was performed by author CJ, a qualitative researcher, based on the constant comparative method, which comprised a systematic approach to data analysis involving (1) open coding of interview transcripts, while comparing codes across transcripts, (2) axial coding where the interplay between

For peer review only

codes was explored to create categories connecting codes together and (3) selective coding of categories to create higher order thematic categories.[15] Data analysis was managed by QSR N6 software. To further increase trustworthiness of the data, investigator triangulation[16] was performed with two other authors (MFP and JDC) reviewing the interpretations of the findings against the participants' quotations.

The study received ethical approval from the Leicester Central National Research Ethics Service Committee, East Midlands Research Ethics Committee (12/EM/0445).

Patient and Public Involvement

The interview topic guide was developed collaboratively with two members of the public with lived experience of Barrett's oesophagus and oesophageal cancer, who virtually commented on a draft version of the topic guide.

Results

We interviewed 18 participants: 16 men and two women, aged 47 to 85 from all six treatment centres. Eleven interviewees participated in the BRIDE trial and seven chose not to. Interviews lasted between 26 to 79 minutes. When discussing factors influencing their decisions whether or not to enrol into the BRIDE trial and a potential future trial comparing endotherapy to surgery, participants mentioned a number of considerations, which have been synthesized into four main themes describing factors intrinsic to: (i) the nature of the trial recruitment process along with the participants' relationship with the trial recruiters and clinical staff, (ii) the participants' perceptions of the design and aim of the study, (iii) participants' motivations for participation balanced against the burden of participation (conditional altruism), and (iv) participants' perceptions of risks of surgery versus endotherapies. These themes are described in more details below:

For peer review only

Nature of recruitment process and role of recruiters

Participants valued the interpersonal skills of the recruiters, irrespective of their grades or professions, placing emphasis on their attitudes and respectful approach. Such attributes translated to a smooth recruitment process to the trial, thereby ensuring that participants did not feel any pressure to participate.

> *"I mean she [the recruiter] was very pleasant …and I think if you've got a disposition like that…it puts you at ease … there's no way that I thought to myself 'I'm pressurised into it' and I fully understood that if ever I wanted to pull out of the study then I can."* (Participant 12)

> *"…it's a very sort of informal situation which I was very comfortable with. I think I'd find it more difficult if I had some very formal doctor. I think the relationship I just feel was being, right from the first I found it very easy to talk to and they were very open in all aspects and they were very inclusive of the patient down there."* (Participant 14)

Participants also placed considerable trust in their clinical team, whom they viewed as the experts in managing their condition. A product of this trust was the belief that, if their clinical team was discussing the option of enrolling into a particular trial, it had to be beneficial for them. Such trust lessened the weight participants placed on written information provided to them as part of the recruitment process, and facilitated their decision-making.

> *"…if he [the consultant] said that he reckoned he could improve the situation, then I was quite happy at just letting him get on with it."* (Participant 15)

For peer review only

"I'd already had some treatments from [Doctor A], and I'd got every confidence in him, so when he came along and said, 'We want to do this and we want to do that', I'd every confidence in him." (Participant 13)

Our findings illustrate the importance of adequate planning when approaching patients for recruitment into trials. On two occasions, participants received information about the trial before being told that they had early cancer, creating significant distress leading to one of the participants declining to be part of the trial. Approaches seen as unconventional by patients, such as being called to a different room or their clinician asking for a nurse or another clinician to be present, caused concern to patients.

"The first time I heard about [the trial] was when I received information through the post… But I hadn't even had my results back from the hospital. Nobody had explained to me what Barrett's Oesophagus was…so to then receive the package, because it was quite a substantial package with consent forms and everything…I wasn't contacted after I had my results or sat down with anybody, I was contacted beforehand and that was a bit alarming…Not just for myself …it was quite upsetting for my wife and my daughter." (Participant 6)

"Before we went to see the surgeon, he [the researcher] wrote a letter to us saying that 'oh you've been diagnosed with this cancer' and he wanted to study, well we didn't know about it… they said 'oh, [Dr B] wants to see you.. and your wife'… we went to see him…and the wife says to him 'OK, if it's bad news, give us the bad news', 'oh no, no', because he'd called in another consultant as well, he called in another consultant and we were wondering… why is he calling another consultant [trial investigator] in, I thought that this meant bad…" (Participant 18)

For peer review only

Participants' perceptions of the study

Participants' understanding of the purpose of the trial varied widely. Participants conflated the aims of the *feasibility* study which they were enrolling in (which was to investigate whether it was possible to conduct a more definitive adequately powered future study) with that of a *definitive* study itself. Those who agreed to participate largely understood that available evidence at the time of the trial showed that both interventions were effective. This belief in clinical equipoise was essential to participants' acceptance for enrolling into the trial and be randomised to one intervention or the other.

> *"People might be a bit reluctant if they think there's a 50/50 chance of them getting a treatment that's more painful or hard to go through, whereas if both options were pretty much the same, which I believe is so in my case, I've got to have treatment so it's a flip of the coin which I get, I don't really mind."* (Participant 9)

> *"Reading on the Internet what it was about, the two procedures and I just got it in my mind that there were roughly the same treatment…"* (Participant 12)

After making their own research into the two interventions, one interviewee did not believe that both treatments were equal, and thereby declined to be part of the trial, choosing instead to receive, what they believed, was the more effective treatment.

> *"I'd done some research…my wife had looked into it as well on the treatment and I couldn't be guaranteed that I'd be given Halo [RFA] treatment. The Halo treatment was a little bit more successful than one of the other treatments that was being offered. So, I then withdrew my consent at the time…"*(Participant 6)

For peer review only

The study design was not always understood by participants, with some believing that their treatment modality was individualised to their needs as opposed to being chosen at random. Such therapeutic misconceptions[17] demonstrated that participants did not always understand the implication of their decisions to enrol in the study and impinged on participants' autonomous decision-making and their ability to give informed consent.

> *"No, he [the clinician] had decided that the gas* [APC] *… would be the best for me… I was quite happy to go along with whatever he suggested."* (Participant 15)

Alarmingly, one participant who had been recruited to take part in the study, did not appear to have any idea what the study was about.

> *"What was the study about, did you understand what was going to go on? [to someone who appeared to be the participant's partner]. … I can't answer your question, I don't know."* (Participant 7)

The implication of non-participation in the trial was also not uniformly understood among participants. Two interviewees believed that active treatment would only be offered to them if they agreed to take part in the trial.

> *"I don't think [the doctor] really had a choice. It wasn't a no choice it's a yes go forward because all the choice was just let it carry on and let it take your life isn't it."* (Participant 2)

> *"I was just approached to say that there were several possible alternative courses of treatment and one of them might involve the BRIDE [trial] effectively. So, I was asked was I willing to consider any of those and I said 'well, of course, because I want to get better, all being well."* (Participant 17)

For peer review only

Conditional altruism

When discussing reasons for enrolling in the trial, interviewees widely reported being motivated by the need to help others and contribute to medical advancement.

> *"As I say, it was simply if it helps somebody in the future, good. 'Cause I'm sure somebody [would have] done exactly the same for the treatment I just had."* (Participant 4)

> *"A letter came through later on and we just thought that we'd help because the National Health, they did a fantastic job on me and basically they saved my life."* (Participant 18)

Such descriptions of altruism and reciprocity were not always unconditional. They were frequently combined with an expectation that participants would also benefit by enrolling in the trial or at least be no worse off. Taking part in the trial was viewed by interviewees as a means of receiving better care by having access to additional information, support and monitoring, which may not be available through standard care.

> *"… I thought maybe I would get monitored more that was my main reason: I really did think that I would be monitored more.."* (Participant 8)

> *"…I work on the principle that hopefully it will help me and whatever findings come out at the end of the trial hopefully it will help somebody else down the line…"* (Participant 11)

> *"That's right and being on the study, I think you get far better treatment as well."* (Participant 16)

For peer review only

As well as considering personal and altruistic motives for participating in the trial, interviewees also gave significance to the amount of personal inconvenience that participating in the trial might entail. Practical concerns included travel, inconvenience to themselves or family, health and financial considerations. Such considerations were largely dependent on the individual's personal circumstances at the time of the approach.

"The main trouble with this study is that it's in [Place 2] and I live in [Place 3], so it's a 40-odd mile trip down. And obviously, having had anaesthetic, I can't drive back. And so one of my daughters had taken me down." (Participant 13)

"The trouble was at the time my late husband was very ill and so it was a question that you know, it was the time factor you know, that I was, I mean OK I wanted to keep myself going for him but also I had to look after him so I couldn't sort of give my full attention to it." (Participant 11)

Risks of surgery

Taking part in the trial was also subject to there being no significant extra treatment or risk to themselves in taking part. Such reflections on risk were fuelled by the fact that both interventions were endotherapies, considered to have similar risk profiles, unlike more invasive surgery.

"…I don't think I had anything to worry about, it [either endotherapy] wasn't going to hurt me or set me back or done any damage to me, it wasn't a risk that way really…they're trying to save you the trauma of a big operation." (Participant 18)

"I think as I've already said, as long as I haven't got to have any extra treatment and it's not going to be much trouble." (Participant 9)

For peer review only

When presented with information about a potential future trial comparing surgery to endotherapy for the management of the same condition, interviewees recognised the differences in the two interventions much more readily. They were not seen as broadly equivalent. Surgery, for many, was seen as riskier and involved additional suffering alongside a longer recovery time. Therefore, participants rejected the notion of clinical equipoise in such a potential future trial.

> *"I think really if it was going to be a flip of a coin, I think that's no way to treat a patient is it."* (Participant 7)

> *"…anything that can be done with an endoscopy must be better than cutting people open. Would have to be at a much greater, later stage and need for people to be opened up."* (Participant 8)

In order to participate in a trial which might involve surgery as intervention when compared to endotherapy, participants felt they would require more information on efficacy and safety. Ironically both of these determinants would not be known before the trial, since a trial comparing surgery with endotherapy in the management of early Barrett's related oesophageal cancer would seek to address these exact answers.

> *"Well, if they [researchers] said either is possible, they [participants]'d want to know which one is the most successful."* (Participant 4)

> *"It depends why they [the researchers] want to do it and what are the advantages of having a surgical procedure as opposed to an endoscopy... But I wouldn't say I wouldn't have it, I would want to know a hell of a lot more about it and what the pros and cons are."* (Participant 1)

For peer review only

Discussion

This study highlights important patient-centred considerations when designing and conducting trials involving endotherapies for early oesophageal cancer. Our findings demonstrate the interactions of recruiters with potential participants before recruitment played a key role in influencing recruitment. Participants valued the respectful and pleasant nature of these interactions, particularly when they were not placed under any duress to participate. Recruitment was facilitated by the dual role played by recruiters who were also often the participants' trusted clinicians. This finding mirrors the results of other interview studies of trial recruiters.[18, 19]

Being both a patient's clinician and a trial researcher can be associated with certain emotional conflicts for the clinical-researcher, which need to be acknowledged.[18, 20] For instance, in an interview study, Donovan et al. highlight challenges expressed by clinicians recruiting into trials around their own treatment preferences,[18] based on their personal opinions. Equally, patient's trust in their clinicians should not be abused to facilitate recruitment. Recruiting clinicians have a duty to be honest and convey both the genuine uncertainty between the safety and effectiveness of the different interventions in the trial (i.e. the clinical equipoise) and be clear on the purpose of feasibility trials, which are designed to inform the design of future more definitive trials. We found both concepts to be poorly understood by certain participants, highlighting potential gaps in the explanation conveyed to them. The literature is also lacking on how recruiters describe and operationalise the concept of equipoise during RCT recruitment.[18, 21]

Our findings highlight the importance of judiciously timed discussions regarding enrolment into studies, in particular where breaking bad news may be involved. We identified

For peer review only

instances when patients were contacted to enrol in research before being told of the diagnosis and management options. We suspect that this situation arose because potential participants for the trial were identified through cancer MDTs by researchers who were not part of their usual clinical teams. While trial recommendation through MDTs have previously been shown to increase recruitment rates,[22] our findings suggest that researchers should not fall into the trap of “recruitment enthusiasm” and ensure that patients are aware of their diagnoses before contact is made. Unexpected deviations from usual outpatient practices when recruiting patients into trials (e.g. having two consultants seeing a patient) also created undue distress.

While altruism did play a factor in influencing recruitment, we found that participants also considered the degree of inconvenience trial participation would entail. McCann et al. previously coined the term “conditional altruism” to describe participants’ willingness to participate in trials to contribute to a greater good so long as they reap some personal benefit from the trial or at least not get harmed.[23] We find that such conditional altruism also entailed an expectation that participation would not be accompanied by personal inconvenience. This finding is particularly relevant for interventions delivered in tertiary centres, as is recommended for the endoscopic management of patients with Barrett’s related neoplasia,[24] and which may involve participants’ time and financial sacrifice, as was the case for the BRIDE trial.

Our findings would suggest that surgery would not be acceptable for participants invited to participate in a trial comparing surgery to endotherapy for early oesophageal cancer unless there was a potential advantage to the more invasive intervention (i.e. surgery) such as higher likelihood of cure, counterbalancing the downsides of increased likelihood of

For peer review only

complications. A recent quality of life survey comparing patients with neoplastic Barrett's oesophagus who had undergone endotherapy to those who had oesophagectomy support this view with the latter group suffering from more long-term symptomatic burden.[25] The issue of recruitment into trials involving surgical interventions is not new, with multiple previous reviews highlighting numerous barriers to such RCTs: the irreversible nature of surgery, strong patient treatment preferences, surgeons' personal opinions and concerns around the idea of randomization.[26-28] We suggest that surgery is only considered an option when compared to endotherapy when there is an established evidence base for the surgical intervention such as high likelihood of cure, counterbalancing the risks of complications. For instance, early oesophageal cancer is associated with certain histological prognostic factors which increase the likelihood of lymphatic spread (such as submucosal invasion to more than 500 microns, or lympho-vascular invasion or poorly differentiated tumours).[29, 30] In such cases, surgery would offer a higher likelihood of cure since local lymph nodes are also removed with the oesophageal resection.[31]

Based on our findings, we suggest some practical considerations to maximising recruitment into future RCTs comparing different endotherapies for the management of patients with early oesophageal cancer. These recommendations may be generally applicable to other RCTs involving invasive interventions, and are summarised in figure 1.

First, there is a need for better coordination between clinical and research teams when contacting potential participants for enrolment. If participants are identified through MDTs, they should only be contacted for participation after being made aware of their diagnoses and treatment options by their clinicians. Clearly, such coordination is made smoother if their own clinician is the trial researcher.

For peer review only

Second, trial recruiters need to acknowledge difficulties in explaining complex concepts such as equipoise and randomisation to patients. Previous trials have used peer feedback to recruiters to improve their own communication and enhanced patient written communication including descriptions of complex trial concepts and interventions in lay-man's terms.[32]

Third, monetary incentives[33], at the very least, to cover subsistence expenses need to be considered. Such incentives are particularly necessary when patients and carers are expected to cover long distances for treatment as part of a trial.

Fourth, we suggest that multi-centre trials need to include opportunities for peer feedback between researchers from different centres during the conduct of a trial to allow sharing of good practice promoting recruitment and discuss challenges.

This study is limited by the small sample although the qualitative methodology used means that the findings are still valid given that sample size in such research is guided by theoretical saturation.[15] Nonetheless, a bigger sample may have led to more themes, as would have inclusion of trial researchers among the interviewees. The study was also conducted before the COVID pandemic. Given the effect of the COVID pandemic on patients' lifestyles and access to care, other themes may have been generated from repeating the study during or after the pandemic. Finally, to allow time for all participants to complete their treatment plans as per trial protocols, interviews were conducted up to a year after participants were initially approached. Such a timescale may have introduced recall bias to the findings of this study.

In conclusion, recruitment and retention of participants into RCTs involving endotherapies is not straightforward. We have demonstrated the value of conducting qualitative research as

For peer review only

part of feasibility trials to inform the design of larger RCTs. We highlight both opportunities and challenges in maximising recruitment to future trials comparing endotherapies and endotherapy versus surgery for the management of patients with early oesophageal cancer.

Based on our findings, we have made some practical recommendations to optimising recruitment and retention in RCTs involving invasive treatments (both endotherapy and/or surgery) for the management of early BO-related neoplasia. These recommendations may be generally applicable to other RCTs involving invasive interventions. Future research should aim to evaluate the views of researchers and clinicians on factors influencing participation in trials comparing different endotherapies for the management of BO-related early oesophageal cancer and the effect of the suggested recommendations on recruitment.

Contributorship

MFP wrote the initial manuscript, edited subsequent versions following feedback from all authors and edited the final version of the manuscript. CJ performed the analysis of the data and provided a data summary. CJ, PB, KR, HB, CS, RH, LBL, HS critically revised and edited all versions. JdC reviewed all versions of the manuscript and edited the final version of the manuscript. All authors approved the final manuscript version being submitted for publication.

Acknowledgement

We would like to thank our two expert patient advisers for their contribution in the design of the study.

For peer review only

Funding statement

This article presents independent research funded by the National Institute for Health Research (NIHR) under its Research for Patient Benefit Programme (grant no. PB-PG-0711-25066).

Competing interests

The views expressed are those of the authors and not necessarily those of the NHS, the NIHR, or the Department of Health. K.Ragunath received educational grants from Erbe and Medtronic. R.Haidry received research infrastructure support from Medtronic, Cook Endoscopy, and Pentax Europe. L.B. Lovat received research infrastructure support from Medtronic. All other authors disclosed no financial relationships relevant to this article.

Ethics approval

The study received ethical approval from the Leicester Central National Research Ethics Service Committee, East Midlands Research Ethics Committee (12/EM/0445).

Data sharing

Data are available upon reasonable request.

Figures

Figure 1 - practical considerations to maximise recruitment into RCTs comparing different endotherapies for the management of patients with early oesophageal cancer

References

References

For peer review only

1. Altman DG. Better reporting of randomised controlled trials: the CONSORT statement. BMJ. 1996 September 07;313(7057):570-1.

2. McDonald AM, Knight RC, Campbell MK, Entwistle VA, Grant AM, Cook JA, et al. What influences recruitment to randomised controlled trials? A review of trials funded by two UK funding agencies. Trials. 2006;7(1):1-8.

3. Treweek S, Lockhart P, Pitkethly M, Cook JA, Kjeldstrøm M, Johansen M, et al. Methods to improve recruitment to randomised controlled trials: Cochrane systematic review and meta-analysis. BMJ open. 2013;3(2).

4. Hennessy M, Hunter A, Healy P, Galvin S, Houghton C. Improving trial recruitment processes: how qualitative methodologies can be used to address the top 10 research priorities identified within the PRioRiTy study. Trials. 2018;19(1):1-5.

5. Que J, Garman KS, Souza RF, Spechler SJ. Pathogenesis and cells of origin of Barrett's esophagus. Gastroenterology. 2019;157(2):349,364. e1.

6. Wani S, Falk G, Hall M, Gaddam S, Wang A, Gupta N, et al. Patients with nondysplastic Barrett's esophagus have low risks for developing dysplasia or esophageal adenocarcinoma. Clinical gastroenterology and hepatology. 2011;9(3):220,227. e1.

7. Bhat S, Coleman HG, Yousef F, Johnston BT, McManus DT, Gavin AT, et al. Risk of malignant progression in Barrett's esophagus patients: results from a large population-based study. J Natl Cancer Inst. 2011;103(13):1049-57.

8. Rastogi A, Puli S, El-Serag HB, Bansal A, Wani S, Sharma P. Incidence of esophageal adenocarcinoma in patients with Barrett's esophagus and high-grade dysplasia: a meta-analysis. Gastrointest Endosc. 2008;67(3):394-8.

9. Oesophageal cancer incidence statistics [Internet].; 2020 [updated 04/; cited 04/03/2021]. Available from: https://www.cancerresearchuk.org/health-professional/cancer-statistics/statistics-by-cancer-type/oesophageal-cancer/incidence#heading-Three.

10. Guidelines on the Diagnosis and Management of Barrett's Oesophagus - An Update. LGD: surveillance or ablation? [Internet].; 2015 [cited Feb/01 2017]. Available from: http://www.bsg.org.uk/images/stories/docs/clinical/guidelines/oesophageal/bsg_barretts_addendum_15.pdf.

11. Manner H, Rabenstein T, Pech O, Braun K, May A, Pohl J, et al. Ablation of residual Barrett's epithelium after endoscopic resection: a randomized long-term follow-up study of argon plasma coagulation vs. surveillance (APE study). Endoscopy. 2014;46(01):6-12.

12. Shaheen NJ, Sharma P, Overholt BF, Wolfsen HC, Sampliner RE, Wang KK, et al. Radiofrequency ablation in Barrett's esophagus with dysplasia. N Engl J Med. 2009;360(22):2277-88.

For peer review only

13. Peerally MF, Bhandari P, Ragunath K, Barr H, Stokes C, Haidry R, et al. Radiofrequency ablation compared with argon plasma coagulation after endoscopic resection of high-grade dysplasia or stage T1 adenocarcinoma in Barrett's esophagus: a randomized pilot study (BRIDE). Gastrointest Endosc. 2019;89(4):680-9.

14. Saunders B, Sim J, Kingstone T, Baker S, Waterfield J, Bartlam B, et al. Saturation in qualitative research: exploring its conceptualization and operationalization. Quality & quantity. 2018;52(4):1893-907.

15. Bryman A. Social Research Methods. New York: Oxford University Press; 2012.

16. Stahl NA, King JR. Expanding approaches for research: Understanding and using trustworthiness in qualitative research. Journal of Developmental Education. 2020;44(1):26-8.

17. Appelbaum PS, Roth LH, Lidz C. The therapeutic misconception: informed consent in psychiatric research. Int J Law Psychiatry. 1982;5(3-4):319-29.

18. Donovan JL, Paramasivan S, de Salis I, Toerien M. Clear obstacles and hidden challenges: understanding recruiter perspectives in six pragmatic randomised controlled trials. Trials. 2014;15(1):5.

19. Donovan JL, de Salis I, Toerien M, Paramasivan S, Hamdy FC, Blazeby JM. The intellectual challenges and emotional consequences of equipoise contributed to the fragility of recruitment in six randomized controlled trials. J Clin Epidemiol. 2014;67(8):912-20.

20. Elliott D, Husbands S, Hamdy FC, Holmberg L, Donovan JL. Understanding and improving recruitment to randomised controlled trials: qualitative research approaches. Eur Urol. 2017;72(5):789-98.

21. Freedman B. Equipoise and the ethics of clinical research. In: Human Experimentation and Research. Routledge; 2017. p. 427-31.

22. McNair A, Choh C, Metcalfe C, Littlejohns D, Barham CP, Hollowood A, et al. Maximising recruitment into randomised controlled trials: the role of multidisciplinary cancer teams. Eur J Cancer. 2008;44(17):2623-6.

23. McCann SK, Campbell MK, Entwistle VA. Reasons for participating in randomised controlled trials: conditional altruism and considerations for self. Trials. 2010;11(1):31.

24. Alzoubaidi D, Ragunath K, Wani S, Penman ID, Trudgill NJ, Jansen M, et al. Quality indicators for Barrett's endotherapy (QBET): UK consensus statements for patients undergoing endoscopic therapy for Barrett's neoplasia. Frontline Gastroenterol. 2019 August 14;11(4):259-71.

25. Reddy CA, Tavakkoli A, Chen VL, Korsnes S, Bedi AO, Carrott PW, et al. Long-Term Quality of Life Following Endoscopic Therapy Compared to Esophagectomy for Neoplastic Barrett's Esophagus. Dig Dis Sci. 2021;66(5):1580-7.

For peer review only

26. Lilford R, Braunholtz D, Harris J, Gill T. Trials in surgery. Br J Surg. 2004;91(1):6-16.

27. Abraham NS, Young JM, Solomon MJ. A systematic review of reasons for nonentry of eligible patients into surgical randomized controlled trials. Surgery. 2006;139(4):469-83.

28. Phelps EE, Tutton E, Griffin X, Baird J. A mixed-methods systematic review of patients' experience of being invited to participate in surgical randomised controlled trials. Soc Sci Med. 2020;253:112961.

29. Herrero LA, Pouw RE, Van Vilsteren F, Ten Kate F, Visser M, van Berge Henegouwen, M I, et al. Risk of lymph node metastasis associated with deeper invasion by early adenocarcinoma of the esophagus and cardia: study based on endoscopic resection specimens. Endoscopy. 2010;42(12):1030-6.

30. Lorenz D, Origer J, Pauthner M, Graupe F, Fisseler-Eckhoff A, Stolte M, et al. Prognostic risk factors of early esophageal adenocarcinomas. Ann Surg. 2014;259(3):469-76.

31. Mariette C, Dahan L, Mornex F, Maillard E, Thomas P, Meunier B, et al. Surgery alone versus chemoradiotherapy followed by surgery for stage I and II esophageal cancer: final analysis of randomized controlled phase III trial FFCD 9901. J Clin Oncol. 2014;32(23):2416-22.

32. Rooshenas L, Scott LJ, Blazeby JM, Rogers CA, Tilling KM, Husbands S, et al. The QuinteT Recruitment Intervention supported five randomized trials to recruit to target: a mixed-methods evaluation. J Clin Epidemiol. 2019;106:108-20.

33. Jennings CG, MacDonald TM, Wei L, Brown MJ, McConnachie L, Mackenzie IS. Does offering an incentive payment improve recruitment to clinical trials and increase the proportion of socially deprived and elderly participants? Trials. 2015;16(1):1-9.

For peer review only

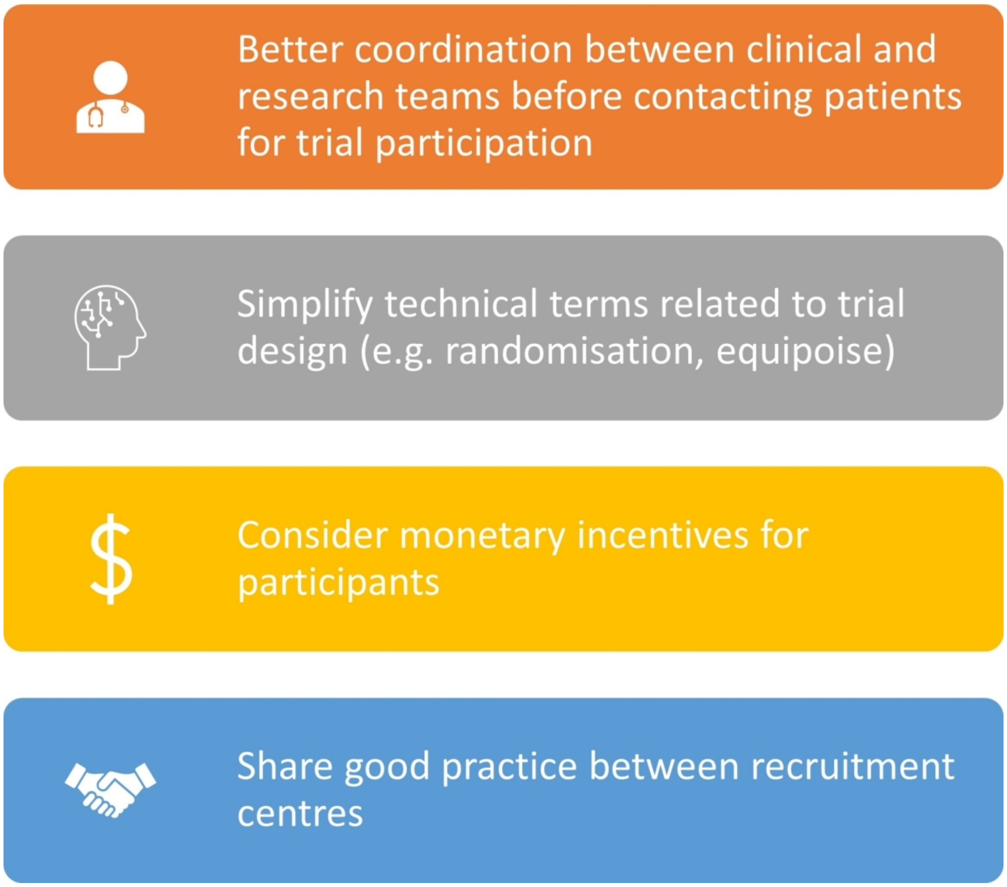

Figure 1 - practical considerations to maximise recruitment into RCTs comparing different endotherapies for the management of patients with early oesophageal cancer

173x152mm (300 x 300 DPI)

Supplementary material A – Interview Topic Guide

This guide is designed to be used flexibly to encourage patients to talk about what matters to them. If the patient brings up an issue not covered here, this will be actively explored. The content is led by the patient, the interviewer's role is to prompt in a way that helps to address the research focus, which is to identify and understand the nature of barriers and enablers to participation in studies of this kind.

The layout of the topic guide may also be amended to suit the individual doing the interviews.

Preliminaries, not necessarily in this order, to be determined by interviewer:

Check that timing of phone call is still convenient.

Introduce interviewer.

Go through information sheet; give opportunity to ask questions, especially confidentiality, anonymity issues, audio recording and transcribing (why it is important).

Confidentiality – what you say will not get back to the center treating you; interviews will be analysed all together, all identifying details removed, and themes summarised. For instance, we might find that several people mention a particular thing – for instance there might be an issue about not feeling rushed – and we will report that; but we would not mention your name or the names of doctors or nurses.

The only situation in which I would break confidentiality is if I heard that someone's life was in danger – this is most unlikely in an interview like this.

For peer review only

Talk through nature of interview – i.e. I am interested in your views so there are no wrong or right answers. I have a list of topics, which is here just to start off the conversation, but what we are really interested in is what matters to you.

Can stop at any time, just ask;

Can say you'd prefer to move on to the next question if there is something that it's not convenient to talk about at the moment

We can take as long as you like, or stop at a particular time – do you have a particular time you need to finish?

Any questions?

We are interested in how people feel about taking part in studies of the sort of treatment you have been having, so we are talking to you because you were asked to take part in a study to do with the treatment of your gullet. Can I check first, is that right?

DECIDING

Some people make up their minds really quickly about taking part in research. Others prefer to weigh things up for longer. How tricky was it for you to decide?

What sorts of things led to your decision?

Prompts

WHO AND HOW recruited

Did it matter WHO asked you, or would you have just said yes/no anyway?

For peer review only

Did it matter HOW it was explained, how much information did you want, what sort of information (explore role of written information, opportunities to talk and ask questions), or would you have said yes anyway …

SHARED OR SOLE DECISION

Many people see this sort of thing as solely their own decision, others make a decision but also like to discuss it with family or friends, how did you feel? (did you talk to partner/family/others about it – tell me a bit more about that)

How did partner/family/others feel about you taking part, did that make a difference to you?

If applicable – how much information did you husband/wife/family want? Who explained it to them? How well did this work out do you think?

PERCEPTION OF AIM AND STRUCTURE OF STUDY

I wondered how it feels to be a patient – were there parts of the study you understood better than others? Did you feel as if you understood what the study aims to do? Can you tell me about that? Were there some things that are harder to understand? (For you, or maybe even if it was OK for you, were there parts that might be hard for other patients). How does it feel to be in the study? How do you think you'd feel if you'd decided against it?

Was there anything that made it easy for you to decide?

Was there anything that made it difficult to decide?

For peer review only

LOOKING BACK:

Looking back, do you feel positive about deciding (not) to take part, or are there some things you are less positive about? (or if you prefer not to look back that's fine, just say so) – What has your treatment been like? How did it feel? Do you know what the treatment was? How was your treatment decided?

If participated: We hope that this didn't happen, but did you at any point feel like dropping out of the study? Tell me about that (what meant that you carried on, how difficult was it, did someone or something make a difference, was there anything someone could have done that would have made that easier etc.)

IF PARTICIPATED: VIEWS ON TAKING PART:

In your view, what have been the good things and the less good things about taking part in the study? Take them in whatever order suits you.

Keep prompting until covered

VIEWS ON TREATMENT:

And in a similar vein, (if not already covered), what have been the good points and the bad points about the treatment you have been receiving? Feel free to mention anything even if it seems minor, because it's important to know what it is like for patients – you are the only expert at being on the receiving end.

For peer review only

Are there any things that you think would help with future studies –?

Things that would help people to decide whether or not to take part

Things that would help people to carry on /not 'drop out',

FUTURE TRIALS:

Now I'd like to ask a few final questions. The treatment centers involved in this study would like to do another piece of research, comparing endoscopic treatment to surgery. We are NOT asking you to take part in this. The reason I am asking you about it is to get your advice because you have some experience of what it's like to be in a study.

Imagine patients were told that there was a need to compare a surgical treatment with an endoscopic treatment because although both seem roughly equivalent, in terms of success, we don't know all their advantages and disadvantages. Taking part would not benefit the patients themselves, but it would provide information that would help in treating people in the future.

How do you think people would feel to be asked whether they were be willing to receive either surgery, or endoscopic treatment, at random (i.e. they would have a fifty fifty chance of each treatment – like deciding by tossing a coin)?

What sorts of things would they want to know before they decided? (explore)

What sorts of things might put them off taking part? (explore)

What sorts of things might mean they were likely to take part? (explore)

For peer review only

Any general messages for people who design and run studies? (i.e. things that would make life easier for those being asked to take part, things that some centers do really well when it comes to research, that other doctors could learn from – or the opposite)

Anything else you want to say about taking part in this type of research? For instance is there anything that makes research into this condition (gullet-related) different to research in other conditions? Tell me a bit about it.

Last - What do you think researchers should be trying to achieve in the future? What should they be looking at, to help with this condition?

Is there anything else you want to say, or to ask me? I cannot answer questions about your particular treatment but I can pass on queries.

Give contact details in case participant wishes to add or retract anything

Thanks

For peer review only

Standards for Reporting Qualitative Research (SRQR)*

http://www.equator-network.org/reporting-guidelines/srqr/

Title and abstract

	Page/line no(s).
**Title** - Concise description of the nature and topic of the study Identifying the study as qualitative or indicating the approach (e.g., ethnography, grounded theory) or data collection methods (e.g., interview, focus group) is recommended	Page 1 Line 2-3
**Abstract** - Summary of key elements of the study using the abstract format of the intended publication; typically includes background, purpose, methods, results, and conclusions	Page 5 Lines 1-17; Page 6 Lines 1-15

Introduction

**Problem formulation** - Description and significance of the problem/phenomenon studied; review of relevant theory and empirical work; problem statement	Start of page 8 to Page 9 Line 5
**Purpose or research question** - Purpose of the study and specific objectives or questions	Page 9, lines 7-15

Methods

**Qualitative approach and research paradigm** - Qualitative approach (e.g., ethnography, grounded theory, case study, phenomenology, narrative research) and guiding theory if appropriate; identifying the research paradigm (e.g., postpositivist, constructivist/ interpretivist) is also recommended; rationale**	Page 9, line 17-18
**Researcher characteristics and reflexivity** - Researchers' characteristics that may influence the research, including personal attributes, qualifications/experience, relationship with participants, assumptions, and/or presuppositions; potential or actual interaction between researchers' characteristics and the research questions, approach, methods, results, and/or transferability	Page 10, line 12-14
**Context** - Setting/site and salient contextual factors; rationale**	Page 10, line 3-4
**Sampling strategy** - How and why research participants, documents, or events were selected; criteria for deciding when no further sampling was necessary (e.g., sampling saturation); rationale**	Page 10, lines 18 to page 11 line 4
**Ethical issues pertaining to human subjects** - Documentation of approval by an appropriate ethics review board and participant consent, or explanation for lack thereof; other confidentiality and data security issues	Page 23, line 12,13
**Data collection methods** - Types of data collected; details of data collection procedures including (as appropriate) start and stop dates of data collection and analysis, iterative process, triangulation of sources/methods, and modification of procedures in response to evolving study findings; rationale**	Page 10, lines 12-17

For peer review only

For peer review only - http://bmjopen.bmj.com/site/about/guidelines.xhtml

**Data collection instruments and technologies** - Description of instruments (e.g., interview guides, questionnaires) and devices (e.g., audio recorders) used for data collection; if/how the instrument(s) changed over the course of the study	Page 10, line 16 and supp mat A
**Units of study** - Number and relevant characteristics of participants, documents, or events included in the study; level of participation (could be reported in results)	Page 11, line 11
**Data processing** - Methods for processing data prior to and during analysis, including transcription, data entry, data management and security, verification of data integrity, data coding, and anonymization/de-identification of excerpts	Page 10, line 18
**Data analysis** - Process by which inferences, themes, etc., were identified and developed, including the researchers involved in data analysis; usually references a specific paradigm or approach; rationale**	Page 10, lines 19- page 11 line 3
**Techniques to enhance trustworthiness** - Techniques to enhance trustworthiness and credibility of data analysis (e.g., member checking, audit trail, triangulation); rationale**	Page 11 – lines 1-3

Results/findings

**Synthesis and interpretation** - Main findings (e.g., interpretations, inferences, and themes); might include development of a theory or model, or integration with prior research or theory	Page 11, line 11 to page 18, line 24
**Links to empirical data** - Evidence (e.g., quotes, field notes, text excerpts, photographs) to substantiate analytic findings	pages 12-18

Discussion

**Integration with prior work, implications, transferability, and contribution(s) to the field** - Short summary of main findings; explanation of how findings and conclusions connect to, support, elaborate on, or challenge conclusions of earlier scholarship; discussion of scope of application/generalizability; identification of unique contribution(s) to scholarship in a discipline or field	Page 19 to page 22 line 11
**Limitations** - Trustworthiness and limitations of findings	Page 22, line 12-22

Other

**Conflicts of interest** - Potential sources of influence or perceived influence on study conduct and conclusions; how these were managed	Page 27, line 19-24
**Funding** - Sources of funding and other support; role of funders in data collection, interpretation, and reporting	Page 27, line 15-17

*The authors created the SRQR by searching the literature to identify guidelines, reporting standards, and critical appraisal criteria for qualitative research; reviewing the reference lists of retrieved sources; and contacting experts to gain feedback. The SRQR aims to improve the transparency of all aspects of qualitative research by providing clear standards for reporting qualitative research.

For peer review only

**The rationale should briefly discuss the justification for choosing that theory, approach, method, or technique rather than other options available, the assumptions and limitations implicit in those choices, and how those choices influence study conclusions and transferability. As appropriate, the rationale for several items might be discussed together.

Reference:

O'Brien BC, Harris IB, Beckman TJ, Reed DA, Cook DA. **Standards for reporting qualitative research: a synthesis of recommendations.** *Academic Medicine*, Vol. 89, No. 9 / Sept 2014
DOI: 10.1097/ACM.0000000000000388

For peer review only
